# The health-economy trade-off during the Covid-19 pandemic: Communication matters

**DOI:** 10.1371/journal.pone.0256103

**Published:** 2021-09-13

**Authors:** Vincenzo Carrieri, Maria De Paola, Francesca Gioia

**Affiliations:** 1 Department of Law, Economics and Sociology, “Magna Graecia” University, Catanzaro, Italy; 2 RWI-Leibniz Institute for Economic Research &Leibniz Science Campus Ruhr, Essen, Germany; 3 IZA, Bonn, Germany; 4 Department of Economics, Statistics and Finance “Giovanni Anania”, University of Calabria, Rende, Italy; 5 Department of Law “Cesare Beccaria”, University of Milan, Milan, Italy; University of Edinburgh, UNITED KINGDOM

## Abstract

How do people balance concerns for general health and economic outcomes during a pandemic? And, how does the communication of this trade-off affect individual preferences? We address these questions using a field experiment involving around 2000 students enrolled in a large university in Italy. We design four treatments where the trade-off is communicated using different combinations of a positive framing that focuses on protective strategies and a negative framing which refers to potential costs. We find that positive framing on the health side induces students to give greater relevance to the health dimension. The effect is sizeable and highly effective among many different audiences, especially females. Importantly, this triggers a higher level of intention to adhere to social distancing and precautionary behaviors. Moreover, irrespective of the framing, we find a large heterogeneity in students’ preferences over the trade-off. Economics students and students who have directly experienced the economic impact of the pandemic are found to give greater value to economic outcomes.

## 1. Introduction

The Covid-19 health emergency prompted governments around the World to adopt unprecedented measures in order to control the spread of the infection. These measures involved the curtailment of basic individual freedoms, which ranged from the total lock-down of economic and social activities to the adoption of precautionary behaviors such as the wearing of masks and the maintaining of interpersonal distance. However, it is quite evident that the efficacy of these measures is strictly related to people’s compliance, which in turn depends on whether or to what extent people agree with them [1]. Self-isolation, social distancing and other precautionary individual behaviors are extremely difficult to impose without people’s willingness to cooperate.

In the absence of financial incentives, a key role for the enforcement of Covid-19 related measures might be played by communication and persuasion policies as “individual compliance has collective benefits, but full enforcement is costly and controversial” [2]. In this paper, we investigate how the framing of the communication of Covid-19 related issues to the public affects individual preferences and, ultimately, their intention to adhere to precautionary behaviors.

We focus on a key aspect of Covid-19 related measures: the inherent health-economy trade-off that they have to deal with. By restricting production and consumption in some sectors, government interventions aimed at reducing the spread of the virus (such as social distancing, lockdowns) produce an immediate negative effect on the economy. On the other hand, in their absence the pandemic could be harsher in terms of a higher number of sick people and, also, more negative effects on the economy. Quantifying costs and benefits of different scenarios, in a situation characterized by a very high level of uncertainty, such as that experienced during the Covid-19 emergency, is particularly complex. Then, in absence of a well-defined counterfactual, it is difficult to assess how much of the economic costs of the pandemic can be considered as indirect costs to be imputed to the policy interventions. Even if it is true that policies characterized by high short-term economic costs can lead to lower costs in the future and, as a consequence, less restrictive policies might not improve but weaken the economy, in the short run individuals perceive especially the economic costs deriving from government restrictions. This is well documented by “reopening protests”, decrying the economic costs of business closure and social distancing, that have been held in many countries from the beginning of the pandemic until today.

In fact, at least in the short run, policy interventions aimed at saving lives tend to produce negative consequences on the economic activity. Quasi-complete lockdown policies implemented by many governments worldwide have mitigated the extent of the spread of the contagion but have also given rise to very considerable short-term economic costs [3–13]. As regards Italy, the estimated cost for each week of closure of all non-essential activities is a reduction in GDP of 0.5–0.75% [14, 15]. The less costly alternatives, e.g. targeted lockdown policies—which often characterize the re-opening phase—make even more explicit the trade-off between health and economic outcomes and such a dilemma can only be expected to persist until a medical response (full vaccination and medical treatments) becomes available. As the health-vs-economy trade-off confronting the social planner is ultimately based on the value that society puts on population health versus short-term economic gains, it is therefore essential to understand how people evaluate health versus economic outcomes and to identify which factors influence their preferences.

We investigate how the framing of communication over both sides of the trade-off affects individual preferences over the policies to be implemented by using a survey field experiment which involved around 2000 students enrolled in a major university in Italy in April 2020. A key element of our research is that of focusing on young individuals. This is not casual. Indeed, while having virtually the same probability of getting the infection, young individuals have also -by far- less severe consequences from it [16, 17]. These two elements (high prevalence but low health consequences) suggest that the behaviors of young individuals are crucial in order to control the diffusion of the pandemic. While data on the violations of Covid-19 measures are not available by age of the offender, the report of the Police department [18] shows that violations happened mostly in areas of ’movida’, i.e. restaurants, pizzerias, fast-food, pubs and bars [19] that are usually frequented by young individuals.

Students involved in our experiment live in the South of Italy, an area that, despite experiencing lower rates of contamination and death, is much weaker from an economic point of view and is endowed with a very poor health system [20–25]. The key question of the survey asked students to reveal their preferences of policies that gave a different weight to the health and economic outcomes of the pandemic. We manipulate the framing of the introductory text of the question associated with the two elements of the trade-off comparing a positive framing which focuses on the protection of health/economic outcomes with a negative framing that presents one or both elements of the trade-off in terms of costs.

We induce the positive framing for health and economic outcomes of the pandemic using the word *protection* and the negative framing using the word *costs*. The choice of the framing—in particular the positive one—is inspired by the language used by politicians and the media during the lockdown in Italy in which expressions such as “health protection” or “health safeguard” have been principally used [26]. For instance, on February 26^th^ (during the initial phase of the epidemic), the Italian Government’s bill including the closure of schools and many economic activities was presented by the ministry of Health as “Actions for the protection of community health” [27]. “La Repubblica”, one of the most widely read Italian newspapers, published 29 articles mentioning the phrase “health protection” during the lockdown phase.

Based on the existing evidence related to the effects of framing [28–30], we hypothesize that students are more likely to choose health—(economy) oriented policies when health (economy) is framed positively instead of negatively. In fact, it is generally found that, when the framing is positive, subjects view the outcomes as gains (showing risk aversion) while, when the framing is negative outcomes are perceived as losses (leading to risk seeking) [31, 32]. A similar hypothesis applies if we consider the link between the two elements of the trade-off. In fact, the worsening of health or economy in the trade-off can be perceived either as a cost or as a loss. The *dead-loss effect* [33, 34] posits that an individual’s subjective state can be improved by framing negative outcomes as costs rather than as losses. In our context, this means that students should be more willing to sustain the costs of the worsening of health or of the economic situation if such a payment is seen as the cost for the protection of the other element of the trade-off, instead of an uncompensated loss. Therefore, our hypothesis is that communicating the trade-off by using a positive framing (*protection*) for one element and a negative framing (*cost*) for the other, instead of negative framing for both, will shift preferences towards the positively framed element and will motivate students to be more willing to sacrifice the negatively framed element.

We find that preferences over the trade-off are related to how the trade-off is communicated. Compared with the framing where both health and economic concerns are expressed as costs, when the trade-off is framed as economic costs to be paid in order to protect against a worsening of health, a large majority of students weigh more the health dimension, deciding to care less about the economic costs to be sustained in return for health protection. Under this framing, 47.36% of students responded that they would prefer policies that consider “extremely” or “very much” the protection of health and “not much” or only a “little bit” the costs for the worsening of the economic situation. On the other hand, under the negative framing, 34.15% of respondents answered that they would prefer policies that consider “extremely” or “very much” the costs for the worsening of health and “not much” or a “little bit” the costs for the worsening of the economic situation. This is consistent with prospect theory predictions—as the positive framing induces risk aversion—and with the *dead-loss effect*. This also supports a large body of empirical evidence that shows that the adoption of healthy behaviors is strongly influenced by the framing strategy [30, 35–37].

Combining health with a positive framing seems to be a low-cost but highly effective communication strategy. In fact, when digging deeper to see whether only audiences with particular characteristics are affected by the positively framed communication, we find that the effect is quite homogeneous, even if women and trustworthy students are found to be particularly reactive.

Regardless of framing, students’ preferences over the health-economy trade-off are highly influenced by several individual characteristics. The field of study (i.e. studying economics) and a pandemic-induced difficult household economic situation affect preferences increasing the weight being given to the economic dimension of the pandemic. On the other hand, students with more highly educated parents, those with altruistic feelings and those who feel more anxious assign more weight to the health dimension. Lastly, when looking at the intention to comply with official advice for self-isolation and precautionary behaviors, we find that intended compliance is higher among students who position themselves more on the health side of the trade-off. Using an instrumental variables approach that exploits the random assignment to the treatments in the framing experiment, we find support for the causality of the relationship that runs from the perception of the trade-off to the compliance with prescribed behaviors.

This paper contributes to the research on framing effects. Beginning with [38], it has been shown that different framings affect the perceived domain of the outcomes thus leading to different choices. The relevance of framing in influencing individual behavioral patterns has been widely documented in a variety of contexts, e.g. in public-sector decision making [28], in health decisions [30] and consumer choices [29]. We complement this literature by analyzing framing effects in a new and previously unexplored setting, i.e. in the midst of an emergency involving two key dimensions of individual well-being such as health and economic outcomes.

We also contribute to the literature in political science that has started to apply behavioral economics insights to the study of political processes (see [39], for a survey). A number of papers has considered the importance of framing in decision-making and applied prospect theory to explain the behavior of governments and leaders in crisis situations [40–44]. [45] show that the decision whom to vote for is strongly influenced by the way policy programs are described. Other works highlight how the political supply side can use some well-known biases, such as loss aversion or the status quo bias, in order to manipulate the evaluation of alternatives. [46] show that whether labor market policies are presented as aiming at lower unemployment or higher employment makes a great difference for public opinion. Other works show that when the outcome of a policy is perceived as a loss, the propensity to take risks to mitigate the situation increases, while when a policy creates benefits that are also perceived as gains, the willingness to take risks to achieve even better results diminishes [41, 42, 47, 48]. Our paper contributes to this literature by investigating a special setting in which—in absence of a direct political competition—citizen’s biased decision-making might be primarily exploited by policy-makers in order to spread pro-social behaviors and thus support the crisis management.

Our paper also speaks to a growing stream of Covid-19 economics literature that is investigating individual perceptions over the health-economy trade-off and compliance with recommended behaviors. For instance, [1] show that how people evaluate health versus short run economic outcomes and compliance with prescribed behaviors depends on the information they receive. They assess public preferences over this trade-off by randomizing information provision on economic and health costs of the pandemic and find that people strongly prioritize health over economy, but these priorities seem to change in predictable ways as the experience of death and income loss unfolds. More importantly, they also find that individuals choosing the maximum valuation of health over economy are more likely to comply with recommended behaviors. Likewise, [49] study the role of cost-benefit considerations in shaping support for mandatory social distancing and stay-at-home measures by varying information on perceived economic costs and health benefits in an experimental setting. However, to the best of our knowledge, no previous paper so far has focused on the role of communication in shaping individual preferences on this trade-off.

Our result that a positive framing on the health side of the trade-off encourages people to worry and care more about health offers valuable insights to public authorities on how to tailor communication after the end of lockdown measures. Framing the policies adopted in terms of “protecting” health could stimulate individuals to place more weight on health concerns and might also positively affect their compliance with precautionary behaviors, helping to contain the spread of the virus. In turn, this could allow policymakers to concentrate more on the economic consequences of the pandemic.

A very preliminary version of this work appeared in [50] and the main insights of our research have been popularized also by [51].

The remainder of the paper is structured as follows. In Section 2 we describe the experimental design, data and balance checks. In Section 3 we discuss our main results. Section 4 is devoted to explore heterogeneous effects across different groups, while Section 5 examines the relationship between health-economy preferences and precautionary behaviors. Section 6 offers some concluding remarks.

## 2. Experimental design, data and balance checks

We study the effect of different communication strategies on individual preferences regarding the trade-off between health and economic outcomes by collecting survey data through a field experiment (randomized controlled trial, RCT). The University of Calabria, in the person of the Rector, approved the study and gave the authorization to collect and analyze data from students anonymously. The invitation to the survey was sent by the administrative office via the institutional email address. Prior to participating, subjects were asked to read a statement about the study, as reported in S1 Appendix. After this statement, they were asked to give or deny consent. By clicking on the consent agreement, they proceeded on to the survey questions.

The survey was submitted on April 20^th^—and remained open until April 25^th^—to about 10,000 students regularly enrolled at the 2^nd^ and 3^rd^ year of the different First Level Degrees, 1^st^ year of the Second Level Degrees and all years of “Lauree a Ciclo Unico” offered by the University of Calabria (61% of them are female; on average they are 22 years old; 29% of them belong to the Department of Social Sciences, 20% to Engineering, 18% to Humanities and 33% to Sciences). Students were randomly assigned to four treatment groups on the basis of their matriculation number. We have firstly divided students into two groups: those with an even matriculation number and those with an odd matriculation number. Then, within each group, we have randomly created two subgroups of equal dimension. Participation in the survey was voluntary and data were collected anonymously. The response rate to our survey was 17.5%.

The four treatment groups were created by manipulating the framing associated with the two elements of the trade-off, thus enabling comparisons between a positive framing which focuses on the protection of health/economic conditions with a negative framing that presents one or both elements of the trade-off in terms of costs. The survey question which was used to induce treatment conditions was the following: “The government is planning the reopening after the temporary self-isolation measures introduced to deal with the coronavirus emergency. At this stage, it is necessary to consider the consequences that each decision has in terms of protection (costs for the worsening) of health—number of infections- and protection (costs for the worsening) of the economic situation. If you were the head of the government, which strategy would you choose?”. Respondents could choose from the following five alternatives: “I would consider extremely the protection (costs for the worsening) of health and not much the protection (costs for the worsening) of the economic situation”; “I would consider very much the protection (costs for the worsening) of health and a little bit the protection (costs for the worsening) of the economic situation”; “I would take into account enough the protection (costs for the worsening) of health and enough the protection (costs for the worsening) of the economic situation”; “I would consider a little bit the protection (costs for the worsening) of health and very much the protection (costs for the worsening) of the economic situation”; “I would consider not much the protection (costs for the worsening) of health and extremely the protection (costs for the worsening) of the economic situation”.

Thus, we design four treatments in a between-subjects design. In the first treatment, *HealthCosts-EconomyCosts* (HC-EC, hereafter), participants are framed the trade-off in terms of costs both for health and for the worsening of the economic condition. In the second treatment, *HealthProtection-EconomyCosts* (HP-EC hereafter), participants are framed the trade-off in terms of protection of health and costs for the worsening of the economic condition. In the third treatment, *HealthProtection-EconomyProtection* (HP-EP, hereafter), both elements of the trade-off are framed in terms of protection while in the fourth treatment, *HealthCosts-EconomyProtection* (HC-EP, hereafter), the choice is between the costs for health and the protection of the economic situation.

In Table 1 we describe the question asking how students would balance health and economic concerns after the end of lockdown measures. In order to make the framing (and thus our treatments) more salient, the same wording is used both in the text of the question and in the text of the possible alternatives among which the students could choose. We report the percentage of students choosing each option under the four different treatments. The HP-EC treatment shifts individual preferences toward policies focusing on health concerns, while under the HC-EP treatment, the option of equally considering both health and economic concerns records the highest percentage of preferences compared to all the other treatments.

**Table 1:** Relative frequencies of responses by treatments.

	HC-EC	HP-EC	HP-EP	HC-EP
	A: costs for the worsening	A: protection	A: protection	A: costs for the worsening
	B: costs for the worsening	B: costs for the worsening	B: protection	B: protection
I would consider extremely the **A** of health and not much the **B** of the economic situation	7.76%	11.42%	7.74%	5.65%
I would consider very much the **A** of health and a little bit the **B** of the economic situation	26.39%	35.94%	26.77%	24.35%
I would consider enough the **A** of health and enough the **B** of the economic situation	63.86%	52.01%	64.16%	68.26%
I would consider a little bit the **A** of health and very much the **B** of the economic situation	1.11%	0.42%	0.66%	1.52%
I would consider not much the **A** of health and extremely the **B** of the economic situation	0.89%	0.21%	0.66%	0.22%

We use responses to the question on how students evaluate the health-economy trade-off to create our dependent variable, *Health-Economy Trade-off*, which is an ordinal variable taking values ranging from 0 (for participants who selected “I would consider not much the protection (costs for the worsening) of health and extremely the protection (costs for the worsening) of the economic situation”) to 4 (for participants who selected “I would consider extremely the protection (costs for the worsening) of health and not much the protection (costs for the worsening) of the economic situation”). Thus, the variable is increasing in terms of the importance given to health concerns.

**Fig 1:**
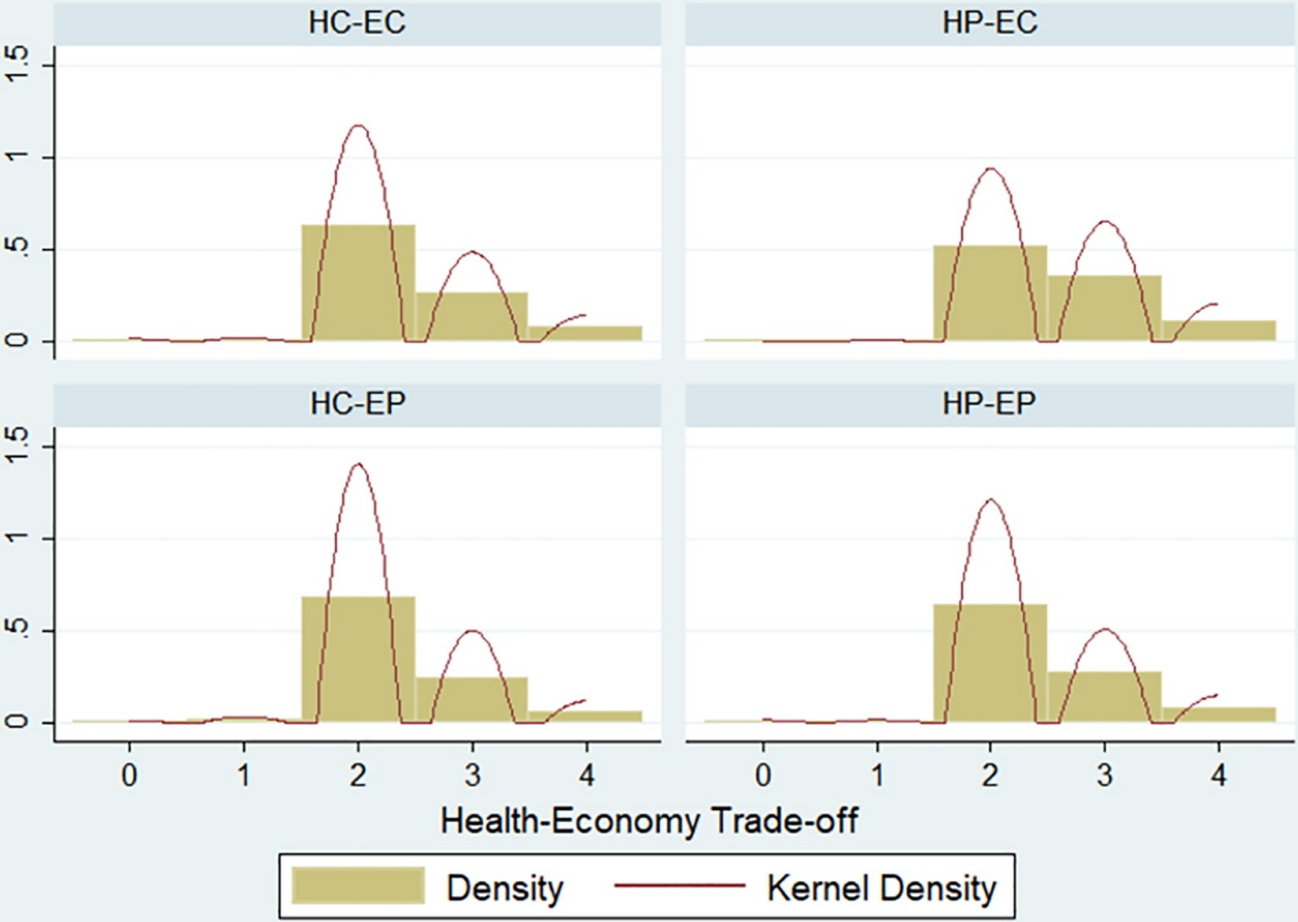
Distribution of responses by treatments.

Fig 1 plots density and Kernel Density estimates of responses to the treatment question by treatment. Two-sample Kolmogorov-Smirnov tests of the equality of distributions show that we can reject the hypothesis that the distribution of the HP-EC treatment is equal to the distribution of the HC-EC treatment (p-value = 0.001), HC-EP treatment (p-value = 0.000) and HP-EP treatment (p-value = 0.001), respectively. Also, Pearson’s chi2 and Fisher’s exact tests show that we can reject the null hypothesis of independence whenever we compare the HP-EC treatment with the other treatments (p-value = 0.001). Comparisons between the remaining pairs of treatments always fail to reject the null hypothesis.

Table 2 reports descriptive statistics of our variables both in the full sample and separately by treatments. *Health-Economy Trade-off* is on average 2.43 in the full sample. It takes on average the value of 2.4 in the HC-EC and HP-EP treatments, the value of about 2.6 in the HP-EC treatment and a lower value (2.3) in the HC-EP treatment.

**Table 2:** Descriptive statistics.

	*All*	*HC-EC*	*HP-EC*	*HP-EP*	*HC-EP*	*F (P-value)*
	(1)	(2)	(3)	(4)	(5)	(6)
Health-Economy Trade-off	2.4286	2.3902	2.5793	2.4027	2.3370	
	(0.6756)	(0.6853)	(0.7030)	(0.6704)	(0.6171)	
Baseline Health-Economy Trade-off	2.090	2.113	2.078	2.071	2.100	0.371
	(0.812)	(0.810)	(0.805)	(0.812)	(0.821)	(0.5423)
**Predetermined characteristics and background**						
Female	0.7086	0.7051	0.7040	0.7212	0.7043	0.2605
	(0.4545)	(0.4565)	(0.4570)	(0.4489)	(0.4568)	(0.6098)
Age	22.3061	22.2927	22.4524	22.2788	22.1957	0.3852
	(2.3514)	(2.2878)	(2.0591)	(2.2131)	(2.7867)	(0.5349)
Parents’ Education	11.7928	12.0477	11.7230	11.6637	11.7413	2.6177
	(3.3168)	(3.0079)	(3.4823)	(3.2946)	(3.4479)	(0.1058)
People/mq	0.0377	0.0384	0.0381	0.0379	0.0362	0.5039
	(0.0228)	(0.0236)	(0.0223)	(0.0266)	(0.0179)	(0.4779)
Sciences	0.3224	0.3060	0.2558	0.2434	0.4848	0.2274
	(0.4675)	(0.4613)	(0.4368)	(0.4296)	(0.5003)	(0.6335)
Humanities	0.1983	0.2239	0.2410	0.2743	0.0543	0.2252
	(0.3988)	(0.4173)	(0.4282)	(0.4467)	(0.2269)	(0.6352)
Engineering	0.1781	0.1885	0.2241	0.1881	0.1109	2.1357
	(0.3827)	(0.3915)	(0.4174)	(0.3912)	(0.3143)	(0.1441)
Social Sciences	0.3012	0.2816	0.2791	0.2942	0.35	1.3112
	(0.4589)	(0.4503)	(0.4490)	(0.4562)	(0.4775)	(0.2523)
**Personality traits**						
Altruist	0.2146	0.2217	0.2051	0.2367	0.1957	0.1706
	(0.4107)	(04159)	(0.4042)	(0.4255)	(0.3971)	(0.6797)
Trustworthy	0.2876	0.2860	0.3044	0.2566	0.3022	0.9106
	(0.4528)	(0.4524)	(0.4607)	(0.4373)	(0.4597)	(0.3401)
Extroverted	0.0561	0.0466	0.0550	0.0575	0.0652	0.7970
	(0.2302)	(0.2109)	(0.2282)	(0.2331)	(0.2472)	(0.3721)
Open to new experiences	0.2228	0.2395	0.2030	0.2677	0.1826	0.5441
	(0.4162)	(0.4272)	(0.4026)	(0.4432)	(0.3868)	(0.4608)
Neurotic	0.1313	0.1109	0.1416	0.1482	0.1239	1.7327
	(0.3378)	(0.3143)	(0.3491)	(0.3557)	(0.3298)	(0.1882)
**Covid-19 health and economic implications**						
Experienced Covid-19	0.1296	0.1441	0.1290	0.1261	0.1196	0.8055
	(0.3360)	(0.3516)	(0.3355)	(0.3323)	(0.3248)	(0.3696)
Parents Unemployed Covid-19	0.2761	0.2550	0.2537	0.3009	0.2957	3.6947
	(0.4472)	(0.4363)	(0.4356)	(0.4592)	(0.4568)	(0.0547)
Depression severity index	9.408	9.226	9.021	9.841	9.559	4.3657
	(5.461)	(5.178)	(5.447)	(5.612)	(5.575)	(0.0368)
Anxiety severity index	13.189	13.208	13.211	13.097	13.237	0.1785
	(3.459)	(3.441)	(3.603)	(3.507)	(3.282)	(0.6727)
**Compliance with prescribed behaviors**						
Compliance PCA	0.067	0.073	0.211	-0.075	0.050	
	(1.692)	(1.559)	(1.296)	(1.803)	(2.023)	
Compliance	648.507	648.213	655.023	641.462	649.020	
	(70.259)	(67.546)	(55.812)	(75.113)	(80.132)	
Observations	1,836	451	473	452	460	

Notes: In columns (1) to (5) we report standard deviations in parentheses. In column (6) we report in parentheses *p*-values for the test of equality of means across treatments.

In order to collect information on students’ baseline preferences towards health and economic concerns, we posed the following question before introducing the treatment (see S1 Appendix for a translation of the survey questions): “Some research shows that the closure of non-essential activities was accompanied in Italy by a reduction of R_t_ (an indicator of the spread of the epidemic) from 8.2 to 0.4. However, each week of non-essential business closures seems to reduce a country’s income and profits by 0.75%. If you were the head of government and the following scenarios were proposed to you for the next two months, which one would you choose: a) No closure, R_t_ = 8.2, Reduction of gross domestic product = 0%; b) Closes ¼ of non-essential activities, R_t_ = 6.15, Gross domestic product reduction = 1.5%; c) Half of non-essential activities closed, R_t_ = 4.1, Gross domestic product reduction = 3%; d) All non-essential activities closed, R_t_ = 0.4, Gross domestic product reduction = 6%”.

The variable *Baseline Health-Economy Trade-off* takes values from 0 (for respondent choosing the option “a”) to 3 (for respondents choosing the option “d”), increasing in the importance given to the health side of the trade-off. It allows us to have a baseline measure of individual preferences that helps to investigate whether treatment effects are homogeneous or are dependent on ex-ante preferences. The average value of the variable in our sample is 2. Baseline and post-treatment preferences for the health-economy trade-off are positively correlated (corr = 0.18, p-value = 0.000).

We have also obtained information on personal characteristics (gender, age, studies, family background, and residence), personality traits, well-being and intention to adhere to social distancing and precautionary behaviors. In Table 1, we report descriptive statistics of each variable both overall and separately by treatment groups. When looking at predetermined characteristics, we see that students are on average 22 years old and about 71% of them are female. As regards their family background, parents have studied on average for 12 years.

As an indicator of students’ personality traits, we included in the survey a question asking students how much they see themselves as a person who is *Altruistic* (21% of the sample), *Trustworthy* (29%), *Extroverted* (6%), *Open to experience* (22%) and *Neurotic* (13%) [52]. Students could choose among 7 alternatives: completely disagree; very much disagree; somewhat disagree; neither agree nor disagree; somewhat agree; very much agree; completely agree. The variables are dummies taking the value of 1 when the answer is “completely agree” and 0 otherwise.

We also collected information on Covid-19 health and economic implications. About 13% of the respondents state that they know someone (relatives, friends or even themselves) who tested positive for the diagnosis of Covid-19 and, for about 28% of students, both parents became unemployed because of the Covid-19 emergency. We also measure students’ psychological conditions including in our survey two modules of the Patient Health Questionnaire (PHQ, a diagnostic tool for mental health disorders used by health care professionals, [53]). On the basis of students’ answers to a depression module and an anxiety module, we build a depression and an anxiety severity scale, respectively. The depression severity scale (calculated by assigning scores of 0, 1, 2, and 3, to the response categories of not at all, several days, more than half the days, and nearly every day, respectively) takes values from 0 to 24 and has an average value of 9.4, while the anxiety severity scale (calculated by assigning scores of 0, 1, 2, 3 and 4 to the response categories it doesn’t match at all; it doesn’t match; neither matches nor does not match; it matches; it matches completely, respectively) takes values from 0 to 20 with an average value of 13.20.

Finally, we asked students to report on a 0–100 range their willingness to comply with the following recommended behaviors: stay at home as much as possible; do not attend social events; wear face mask; stay at least two meters from other people; wash hands frequently; stay at home with symptoms of coronavirus; avoid hugs and handshakes. Using responses to these questions, we built two measures of compliance to these behaviors. First, we create a variable—named *Compliance PCA*—through a Principal Component Analysis of each of the seven questions on prescribed behaviors. As an alternative variable, we construct a “count” measure of compliance, summing up the values of the seven variables, and obtaining an indicator that ranges between 0 (when all the seven variables take the value of 0) and 700 (when all the seven variables take the value of 100). We adopt this approach as, in practice, the incidence of compliance is highly correlated across the different behaviors. For instance, the correlation between the intention to “Stay at home when sick” and “Wash your hands frequently” is equal to 0.557, p-value = 0.000, while the correlation between “Avoid hugs and handshakes” and “Stay at least 2 meters from other people” is equal to 0.563, p-value = 0.000. The average value of the variable is 648, it ranges from 641 in the HC-EP treatment to 655 in the HP-EC treatment. These high levels of compliance with recommended behaviors are consistent with findings reported by [54] who rely on a representative survey of Italian adults conducted during the initial phase of the Covid-19 pandemic.

To investigate the effects that the four treatments produce on individual outcomes we need four comparable groups. The last column of Table 2 reports p-values of tests of equality of variables’ means among treatments. Treatment groups are evenly balanced on a large number of covariates (with the exclusion of *Parents Unemployed Covid-19*) and data regarding predetermined characteristics show that we are unable to reject the hypothesis that the randomization was successful in creating comparable treatment groups in respect of observable characteristics in the subsample of students submitting their responses to the survey questions. We have also tested the equality of variables means for each possible pair of treatments. We find that treatments are always equally balanced in terms of age and gender but sometimes they present differences in the distribution of the field of study. For this reason, in our estimates we control for field of study dummies. Also, if we compare predetermined characteristics of respondents with those of the average student population we find that our sample is quite representative of the student population, along the dimensions of age and field of study while, due to a higher response rate, women are slightly over-represented (61% of students included in the survey are female).

## 3. Communication and preferences for health and economy: Main results

In this section we carry out an econometric analysis to investigate whether being assigned to the four different framings adopted in our experiment induces students to balance differently health and economic outcomes.

We estimate several specifications of the following simple model:

$$Health-Economy\;Trade-off_{i}=\beta_{0}+\beta_{1}{\left(HP-EC\right)}_{i}+\beta_{2}{\left(HC-EP\right)}_{i}+\beta_{3}{\left(HP-EP\right)}_{i}+\beta_{4}X_{i}+\beta_{5}F_{i}++\beta_{6}BaselineHealth-EconomyTrade-off_{i}+\beta_{7}W_{i}+\beta_{8}Z_{i}+u_{i}$$ (1)

where the vector *X*_*i*_ includes individual pre-determined characteristics (gender, age, field of study, etc.), *F*_*i*_ includes family background variables (parents’ education, etc.), *W*_*i*_ includes controls for Covid-19 health and economic implications (parents’ employment, experience with Covid-19, psychological conditions), *Z*_*i*_ is a set of variables measuring current personality traits, and *u*_*i*_ is the error term.

In this setting, *β*_*1*_ is the difference between HP-EC and HC-EC (that is the treatment effect of framing health in terms of protection instead of costs) in the propensity to favor policies that give greater weight to health concerns arising from the spread of Covid-19. Positive values of *β*_*1*_ suggest that, in the management of the reopening after the lockdown measures, communicating the trade-off using for health a positive framing which focuses on protective strategies—instead of a negative framing based on costs—increases students’ concerns for the health consequences of the pandemic. A similar interpretation holds for *β*_*2*_ and *β*_*3*_ that represent the effect induced by the other two treatments, HC-EP and HP-EP, respectively, with respect to the framing HC-EC.

Our hypotheses are the following:

- H1: *β*_1_>0, that is, the use of a positive framing (protection) for health induces students to associate a greater weight to health in the trade-off, being both more risk averse on this domain and more inclined to bear higher economic costs as they are seen as a payment needed in order to protect health;
- H2: *β*_2_<0, that is, the use of a positive framing (protection) for economic outcomes increases the weight of economic outcomes in the trade-off for the same reasons as above;
- H3: *β*_3_≥0, that is when both elements of the trade-off are framed in terms of protection; either they should carry the same weight or, given the strong health concerns under a pandemic, the protection of health may carry more weight.

In Table 3 we report estimation results of several specifications of model (1). We estimate an Ordered Probit Model to study the effect of the assigned treatment condition on the probability of students giving greater consideration to health concerns in policy decisions. Since the dependent variable increases with the importance associated with health concerns, positive coefficients suggest the likelihood of preferences being more shifted toward health concerns. In all the regressions, standard errors (corrected for heteroscedasticity) are reported in parentheses.

**Table 3:** The impact of communication on preferences for policies aimed at managing the Covid-19 crisis.

	*Health-Economy Trade-off*					
	(1)	(2)	(3)	(4)	(5)	(6)
HP-EC	0.3241***	0.3390***	0.3445***	0.3617***	0.3630***	0.3660***
	(0.0771)	(0.0772)	(0.0780)	(0.0787)	(0.0786)	(0.0788)
HC-EP	-0.0998	-0.1191	-0.1152	-0.1221	-0.1177	-0.1140
	(0.0791)	(0.0808)	(0.0814)	(0.0817)	(0.0820)	(0.0821)
HP-EP	0.0251	0.0381	0.0378	0.0442	0.0611	0.0572
	(0.0799)	(0.0799)	(0.0804)	(0.0804)	(0.0808)	(0.0808)
Female		-0.0200	-0.0204	-0.0225*	-0.0242*	-0.0246*
		(0.0131)	(0.0132)	(0.0130)	(0.0130)	(0.0129)
Age		-0.0125	-0.0114	0.0012	-0.0885	-0.0913
		(0.0667)	(0.0666)	(0.0667)	(0.0724)	(0.0722)
Sciences		0.2502***	0.2461***	0.2122***	0.2159***	0.2172***
		(0.0690)	(0.0694)	(0.0705)	(0.0713)	(0.0715)
Humanities		0.1125	0.1118	0.0767	0.0719	0.0654
		(0.0844)	(0.0845)	(0.0852)	(0.0859)	(0.0859)
Engineering		0.0725	0.0615	0.0100	0.0257	0.0300
		(0.0870)	(0.0880)	(0.0883)	(0.0881)	(0.0885)
Parents’ Education			0.0211**	0.0219***	0.0205**	0.0206**
			(0.0084)	(0.0085)	(0.0086)	(0.0086)
People/mq			2.8744**	2.8106**	2.8528**	2.7569**
			(1.3024)	(1.2939)	(1.3067)	(1.3109)
Baseline Health-Economy Trade-off				0.2644***	0.2512***	0.2508***
				(0.0372)	(0.0370)	(0.0369)
Parents Unemployed Covid-19					-0.1284**	-0.1267**
					(0.0632)	(0.0634)
Experienced Covid-19					0.0464	0.0479
					(0.0848)	(0.0850)
Anxiety severity scale					0.0423***	0.0419***
					(0.0104)	(0.0104)
Depression severity scale					-0.0079	-0.0100
					(0.0060)	(0.0061)
Altruist						0.1507*
						(0.0796)
Trustworthy						-0.0589
						(0.0670)
Extroverted						0.0464
						(0.1285)
Open new experiences						0.0111
						(0.0775)
Neurotic						0.0164
						(0.0930)
Province of Residence FE	NO	NO	YES	YES	YES	YES
Observations	1836	1836	1836	1836	1836	1836
PANEL A						
	*p*-value					
H_0_: HP-EC = HP-EP	0.000	0.000	0.000	0.000	0.000	0.000
H_0_: HP-EC = HC-EP	0.000	0.000	0.000	0.000	0.000	0.000
H_0_: HP-EP = HC-EP	0.109	0.053	0.060	0.042	0.030	0.037
PANEL B						
H1						
H_0_: HP-EC< = 0, H_1_: HP-EC>0	0.000	0.000	0.000	0.000	0.000	0.000
H2						
H_0_: HC-EP> = 0, H_1_: HC-EP<0	0.103	0.070	0.079	0.068	0.076	0.082
H3						
H_0_: HP-EP> = 0, H_1_: HP-EP<0	0.623	0.684	0.681	0.709	0.775	0.761

Ordered Probit Estimates.

Notes: Standard errors (corrected for heteroscedasticity) are reported in parentheses. The symbols ***, **, * indicate that the coefficients are statistically significant at the 1, 5 and 10 percent level, respectively.

As shown in column (1), where we do not include controls, we find that, compared with the HC-EC treatment, the HP-EC framing induces students to choose a policy that gives greater consideration to health issues. Thus, our data fail to reject hypothesis H1: when the trade-off is communicated as health protection versus costs for the worsening of the economic situation, instead of framing both health and economy as costs, respondents perceive the worsening of the economic situation as a cost allowing for protection against the worsening of health, instead of as an uncompensated loss, and are therefore more willing to sustain it. This is confirmed by the test on the coefficient reported at the bottom of the Table (Panel B) showing that we can reject the hypothesis that the coefficient is smaller than or equal to zero at a 0.01 significance level. The shift in preferences that favor policies that mainly focus on health issues produced by the HP-EC treatment is statistically significant also when compared with the other different types of framing used in our experiment, as shown in Panel A.

As regards hypothesis H2, we find evidence for a negative effect of the positive framing associated with economic outcomes on the preference for health-oriented policies. The test of our hypothesis reported in Panel B shows that, in all estimates but the first one, we can reject the hypothesis that the coefficient is greater than or equal to zero at a 0.10 significance level. Finally, when looking at the HP-EP treatment (H3), we find a positive but not statistically significant from zero coefficient. This is consistent with the test reported in Panel B and would suggest that framing both elements of the trade-off in terms of protection is the same as using the framing “costs” and, even under a pandemic, the protection of health does not carry significantly more weight when joined with the protection of the economic situation. When comparing HP-EP with the remaining treatments (Panel A) we find that the HP-EC treatment generates a significantly bigger shift in preferences that favor policies that mainly focus on health while the effect of the HP-EP treatment is significantly different from the HC-EP treatment (which induces a shift towards economy centered policies) in all but the first specification.

These results remain qualitatively unchanged when we add controls for age, gender and field of study (column 2) and when we also add controls for family background and province of residence fixed effects (column 3). In column (4) we include among controls our measure *Baseline Health-Economy Trade-off*, which is positively correlated with preferences for a health-centered policy, but does not affect the influence produced by our treatment conditions. No relevant changes are found also when we add, among regressors, proxies for individual exposure to the Covid-19 emergency both in terms of health and economic outcomes (column 5) and when we control for individual personality traits (column 6).

The impact of the HP-EC treatment is sizeable. When looking at average marginal effects for the specification including all the control variables (column 6) we find that when the trade-off is expressed in terms of protection of health and costs for the worsening of the economic situation—instead of in terms of costs for both health and the economy—students are about 0.45% less likely to choose the policy giving the greatest weight to the economic situation; about 0.78% less likely to choose the policy considering a little bit health and very much economic outcomes; 11.8% less likely to choose the intermediate policy; 7.7% more likely to choose the policy considering very much health and a little bit economic outcomes and about 5.2% more likely to choose the policy that gives greatest weight to health concerns.

As regards control variables, we find that the field of study reveals different preferences and that students enrolled in scientific disciplines tend to prioritize health concerns compared with students enrolled in economics and social sciences and engineering. There is also an important difference in terms of socio-economic background; students who have more highly educated parents and who live in larger houses show a preference for policies that tend to favor health protection. Since both parental education and floor space per person are usually associated with the economic conditions of the family, the result shows that those who come from contexts of greater economic distress tend to give greater weight to the economic costs of the pandemic while for students having better-off families the trade-off may be less salient [55]. This is also confirmed by the fact that students with parents who lost their jobs due to the emergency tend to express themselves more favorably towards a compromise that takes due account of the economic costs of the crisis. On the other hand, students who are particularly anxious, due to the Covid-19 emergency, are more favorable to policies more focused on health issues. Finally, those who describe themselves as altruistic also tend to prefer health-centered policies.

In S2 Appendix, we show the robustness of our results to Ordered Logit and Multinomial Logit estimates. As suggested by the distribution in Table 1, Multinomial Logit estimates show that the effect of the HP-EC treatment is statistically significant for the two categories referring to more health centered policies.

To check the robustness of our results, we have also created, as an outcome variable, a dummy taking the value of 1 for students who report preferences for policies that give ‘very less’ or ‘less’ relevance to the economic costs of the crisis and 0 otherwise. Probit estimates are qualitatively very similar to those discussed above. The only difference concerns the HC-EP coefficient that now is more precisely estimated but still typically not statistically significant at conventional levels. In addition, we have created another ordinal variable taking values ranging from 0 to 2 where 0 and 2 are participants selecting the two extreme options (respectively 0 = participants who selected “I would consider not much the protection (costs for the worsening) of health and extremely the protection (costs for the worsening) of the economic situation”; 2 = participants who selected “I would consider extremely the protection (costs for the worsening) of health and not much the protection (costs for the worsening) of the economic situation”) while the value of 1 is assigned to all participants selecting the intermediate options. Finally, since the middle option is the “neutral” response (which could be interpreted as a “don’t know” answer for many respondents) we have also tested our results to the exclusion of such respondents from the sample. Our evidence of a positive and statistically significant effect of the HP-EC treatment holds in both cases. Results are not reported but are available upon request.

## 4. Heterogeneous impact of framing

In the previous sections we have seen that a simple communication strategy, that positively frames the health side of the health-economy trade-off arising from the current health emergency, impacts on students’ preferences towards the trade-off. Nonetheless, communication takes place in different contexts and is directed to different audiences, who might be more or less reactive to how messages are framed. Then, in this section, we investigate if individual characteristics, such as gender, economic and social background, personality traits, experiences and beliefs, can amplify or nullify the impact of framing.

With this aim, we analyze whether our treatments have produced heterogeneous effects across the three sets of controls that we have considered in the previous analysis (predetermined characteristics and background; personality traits; Covid-19 health and economic implications) and whether the impact is related to individual baseline preferences. This would suggest in which circumstances framing can be effectively used to try to build up consensus towards certain types of policies. For each control that we consider, we report bar graphs showing the difference in the level of our indicator *Health-Economy Trade-off* between each of our treatments and the reference treatment HC-EC, separately by category, and 95% confidence levels, based on the estimates of a seemingly unrelated regression equations (SURE) model reported in detail in Tables C1-C4 in S3 Appendix. For robustness, in Tables C5-C8 in S3 Appendix, we also report the estimates of a model with interaction terms and p-values obtained applying the Sidak’s and the Holm’s adjustment for multiple testing. Albeit the two models lead to very similar conclusions, in what follows, we will refer mostly to the interaction model to assess the statistical significance of the heterogeneous analyses.

**Fig 2:**
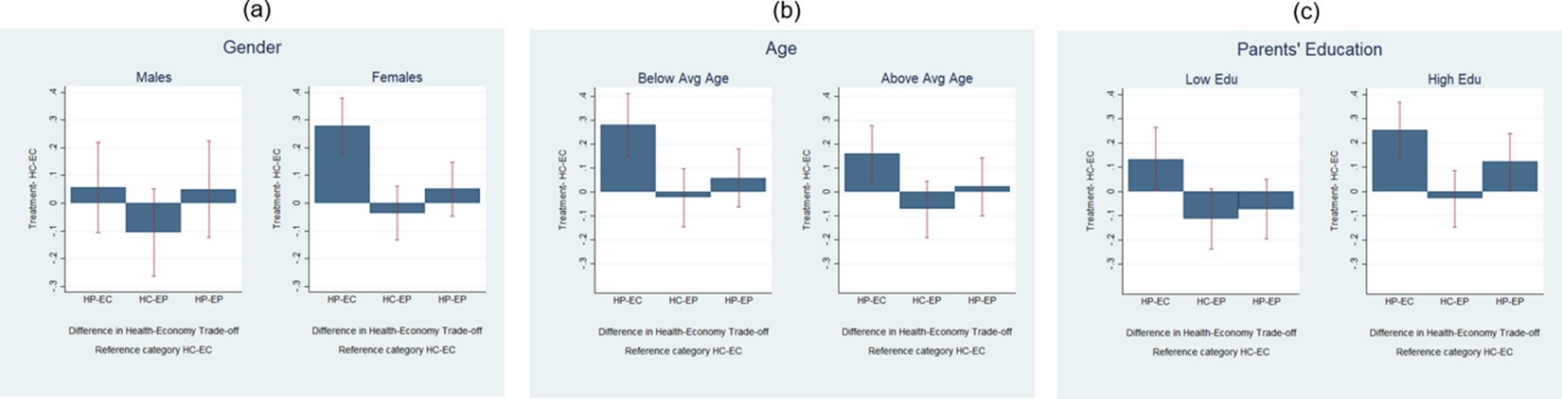
Predetermined characteristics and background.

In Fig 2 we look at two predetermined characteristics and an indicator of family background. Fig 2(A) focuses on gender and reports mean values of *Health-Economy Trade-off* for each treatment, separately for men and woman. We can see that for both genders the variable *Health-Economy Trade-off* has the highest mean value when health is associated with a positive framing; however, females show a large and statistically significant shift in preferences over the health-economy trade-off towards health-oriented policies when health is framed using the positive word “protection”. Differences across gender are statistically significant for what concerns the comparison between HP-EC treatment but not statistically significant when considering the other treatments (Table C5 Column 1 in S3 Appendix). This evidence of females’ higher sensibility to the positive framing for health is in line with [56] who find that on average females are more likely to perceive Covid-19 as a very serious health problem and to agree and comply with precautionary behaviors.

In Fig 2(B) we look at *Age* and split the sample into students with an age higher than (or equal to) 22 (the average) and students younger than 22 years old. Again, for both categories it emerges that there is a positive effect of the HP-EC treatment; nonetheless, the differences are not statistically significant across age groups for all treatments (Table C5, Column 2 in S3 Appendix). In Fig 2(C) we split our sample according to parents’ education (above and below the median). We find that the HP-EC treatment shifts the preferences towards health for both groups in a significant way. However, differences across groups in the effect of the treatment are not statistically significant when using Sidak- Holm adjustment (Table 5, Column 3). Interestingly, compared with the HC-EC treatments, students with more highly educated parents when exposed to HP-EP treatment increase their favor towards policies focusing on health concerns quite substantially, albeit in a not statistically significant way (Table C5, Column 3 in S3 Appendix) and this can be due to a high variance in the responses. Heterogeneity across the other dimensions, such as the number of squared meters available for each person in the house, does not produce any significant effect.

**Fig 3:**
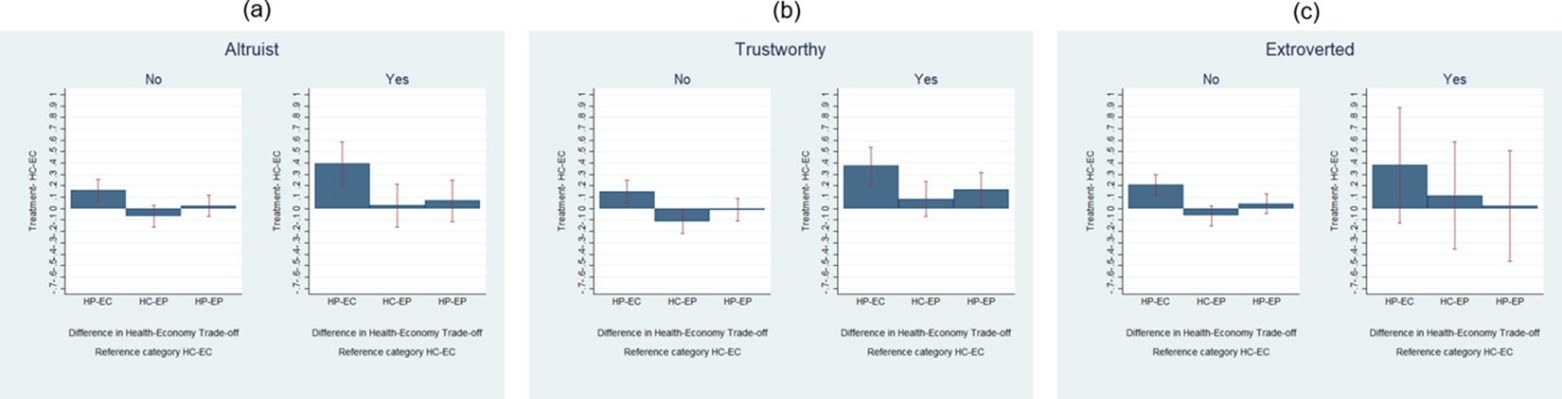
Personality traits.

In Fig 3 we investigate whether students’ reactions to how communication is framed are related to their self-reported personality traits. We here consider only altruism, trustworthiness and extroversion because students claiming to be opened to experience and neurotic have preferences very close to those not self-defining likewise. Fig 3(A) looks at altruistic students who, according to estimates shown in Table 3, tend to prefer policies that focus on the health side. The graph clearly shows a significantly stronger effect of the HP-EC treatment on these students compared to others who did not see themselves as particularly altruistic, albeit this difference is not statistically significant across the two groups (Table C6, column 1 in S3 Appendix). Likewise, when looking at *Trustworthy* students (Fig 3(B)) we find that they report preferences significantly shifted towards health-oriented policies (higher values of *Health-Economy Trade-off*) in the HP-EC treatment compared with the HC-EC treatment. Compared with other students, they also seem to favor more health in all treatments except for the HC-EC treatment and this difference is also statistically significant for HP-EC and HC-EP (Table C6, column 2 in S3 Appendix). This result is important since it shows that trust can be an important lever to influence preferences during an emergency situation and build up consensus towards certain types of policies. Finally, when looking at extroversion in Fig 3(C), it emerges that students who consider themselves as extrovert are more influenced by the HP-EC treatment than their not extrovert counterparts but in a not statistically significant way (Table C6, column 3 in S3 Appendix).

**Fig 4:**
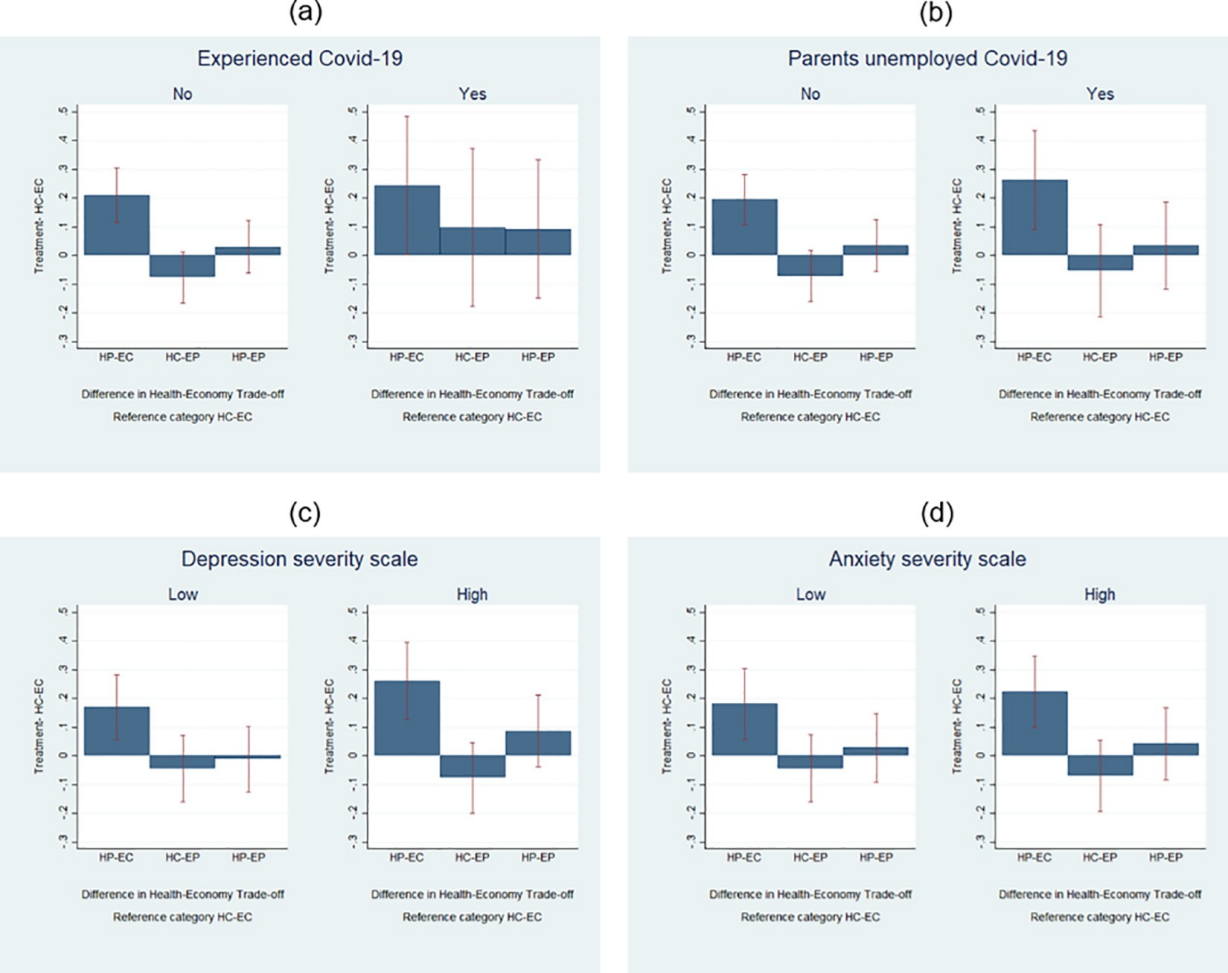
Covid-19 health and economic implications.

We have also examined the role of students’ exposure to the Covid-19 emergency in terms of both health and economic implications. Firstly, we have considered whether students more directly exposed to Covid-19, because relatives or friends tested positive to the virus, are less or more influenced by framing. Fig 4(A) shows that preferences are overall similar across students for what concerns HP-EC treatment. The only relevant difference is that individuals who more closely experienced the epidemic show preferences more shifted towards health policies across all treatments (in comparison with HC-EC), while students who have not closely experienced the epidemic seem to have preferences more in favor of policies that tend to limit the impact of the crisis on the economy when not exposed to HP-EC treatment. Differences across these groups are, however, not statistically significant (Table C7, column 1 in S3 Appendix). Then, to consider personal exposure to the economic crisis, we have split the sample according to whether or not students’ parents have lost their jobs due to the pandemic. We find similar preferences in all the treatments.

Additionally, as the way in which individuals react to the communication messages they receive also depends on their psychological conditions, in Fig 4(C) and 4(D) we split our sample considering the depression and anxiety severity scales we have described in Section 3. The figures confirm the effect of a positive framing for health concerns. In particular, we find that for students who feel more depressed, the increase in preferences for more health-oriented policies when the trade-off is expressed in terms of protection of health and costs for the worsening of the economic situation—instead of in terms of costs for both health and the economy—is almost twice as large as the effect found for students in better psychological conditions. Differences across these groups are anyway not statistically significant when using Sidak-Holm adjustment (Table C7, columns 3 and 4 in S3 Appendix).

**Fig 5:**
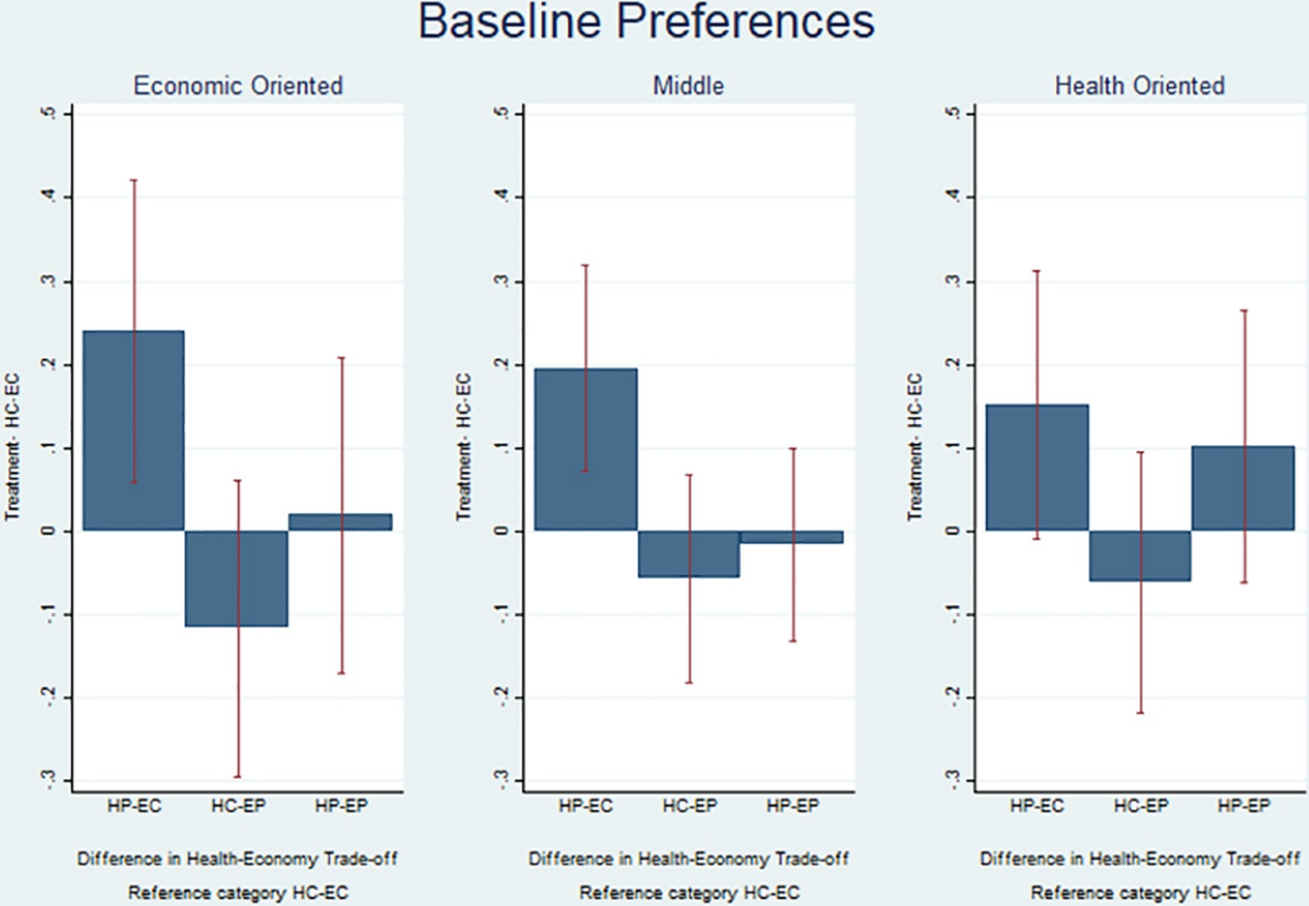
Baseline preferences.

As a last heterogeneous effect analysis, we also look at whether the framing has a differential effect among students with different baseline preferences over the health-economy trade-off. Fig 5 compares the average value of *Health-Economy Trade-off* for students who, before the treatment, indicated to having economic oriented, middle or health-oriented preferences, respectively. The graph shows that albeit baseline and ex-post preferences are positively correlated, the treatment is able to shift preferences towards Health-Centered Preferences quite importantly. Indeed, when the positive framing is used for the health side of the trade-off, even those with ex-ante more economic oriented preferences shift towards policies that assign higher value to health and indeed these students are those experiencing the highest treatment effect as compared with the HC-EC treatment. We do not find a specular effect when using the positive framing for the economic side of the trade-off. Differences across these groups are anyway not statistically significant when using Sidak-Holm adjustment (Table C8 in S3 Appendix).

All in all, our analysis shows that, a simple and zero cost communication strategy that associates a different framing to the two sides of the trade-off has a widespread effect with very few statistically significant differences across different audiences. The only noteworthy exceptions are represented by females and trustworthy students who seem to react significantly more to the HP-EC treatment with respect to their counterparts.

## 5. Communication and compliance with prescribed behaviors

Our analysis so far has shown that communication style affects individual preferences over the health-economy trade-off of Covid-19 related policies. In this section, we take a further step by looking at how preferences over the trade-off correlate with intentions to adhere to behaviors that have been suggested as useful tools to limit the spread of the epidemic (i.e. wash hands; avoid touching eyes, nose, mouth; stay at least two meters from other persons, stay at home with symptoms of coronavirus). These are measured by the two proxies described in Section 2, *Compliance PCA* and *Compliance*. Even if, as discussed by [57, 58], self-reported measures of compliance with prescribed behaviors might suffer of desirability bias, this would not qualitatively affect our results if the desirability bias is not differentiated in relation to the variable of interest, *Health-Economy Trade-off*, and ultimately in relation to our treatments (which, as discussed below, are used as instruments to deal with potential endogeneity in preferences over the health-economy trade-off).

In Table 4 we report OLS estimation results investigating the relationship between intention to adhere to prescribed behaviors and preferences for health centered policies (*Health-Economy Trade-off*). In odd columns we consider as the outcome variable *Compliance PCA*, while in even ones we use *Compliance*. As shown in columns (1) and (2), without controls, we find that *Health-Economy Trade-off* is positively and significantly correlated with both measures of compliance. The same results hold true when we add among the regressors individual and family characteristics and our measure of baseline preferences (columns 3 and 4) and the full set of controls (columns 5 and 6). As regards control variables, we find that women are more likely to follow precautionary behaviors. A positive correlation is found also for *Baseline Health-Economy Trade-off*, *Parents’ education*, *Anxiety severity index*, *Trustworthy*.

**Table 4:** Preferences for health centered policies and intention to adhere to prescribed behaviors.

	Compliance PCA	Compliance	Compliance PCA	Compliance	Compliance PCA	Compliance
	(1)	(2)	(3)	(4)	(5)	(6)
Health-Economy Trade-off	0.2873***	13.7449***	0.2364***	11.4819***	0.2062***	10.2431***
	(0.0736)	(2.9359)	(0.0700)	(2.7974)	(0.0680)	(2.7020)
Female			0.5198***	22.0261***	0.4256***	18.5197***
			(0.0865)	(3.6371)	(0.0981)	(4.0564)
Age			-0.0208	-0.7726	-0.0217	-0.7953
			(0.0222)	(0.8656)	(0.0220)	(0.8540)
Science			0.0252	1.9127	0.0546	3.1757
			(0.1068)	(4.4096)	(0.1050)	(4.3413)
Humanities			0.1615*	4.7624	0.1735*	5.2562
			(0.0967)	(4.2380)	(0.0971)	(4.2545)
Engineering			0.0939	5.9801	0.1435	8.2332*
			(0.1177)	(4.8936)	(0.1154)	(4.7922)
Parents’ Education			-0.0021	-0.1973	0.0005	-0.0999
			(0.0131)	(0.5466)	(0.0133)	(0.5541)
People/mq			0.7468	45.7110	0.8292	50.3332
			(2.1032)	(82.9551)	(2.1330)	(83.4950)
Baseline Health-Economy Trade-off			0.1959***	8.8720***	0.1805***	8.1802***
			(0.0549)	(2.2563)	(0.0553)	(2.2678)
Parents Unemployed Covid-19					-0.0384	-2.4223
					(0.0905)	(3.7195)
Experienced Covid-19					-0.0156	-0.3317
					(0.1192)	(5.0040)
Anxiety severity scale					0.0575***	2.2620***
					(0.0131)	(0.5516)
Depression severity scale					-0.0236***	-1.0864***
					(0.0090)	(0.3651)
Altruist					0.0448	2.1857
					(0.1088)	(4.4941)
Trustworthy					0.2800***	13.1387***
					(0.0808)	(3.4611)
Extroverted					-0.2278	-9.5914
					(0.1994)	(8.3191)
Open new experiences					-0.1387	-8.1236*
					(0.1019)	(4.3644)
Neurotic					-0.0150	0.8255
					(0.1283)	(5.2747)
Prov. of Residence FE	NO	NO	YES	YES	YES	YES
Observations	1836	1836	1836	1836	1836	1836

OLS Estimates.

Notes: Standard errors (corrected for heteroscedasticity) are reported in parentheses. The symbols ***, **, * indicate that the coefficients are statistically significant at the 1, 5 and 10 percent level, respectively.

The positive correlation between *Health-Economy Trade-off* and individual compliance shown in Table 4 indicates that students with preferences for health-centered policies are generally more likely to have an intention to adhere with prescribed behaviors. However, this does not imply causation since it is possible that unobserved factors associated with both the perceived trade-off and compliance cause a spurious correlation between these two variables. Thus, in order to gain a better understanding of the extent to which preferences regarding the health-economy trade-off causally affect adherence with prescribed behaviors, following [49], we adopt an instrumental variable approach. This strategy strongly relies on the availability of a valid instrument, that is a variable that (1) affects the endogenous variable (relevance) but (2) shows no independent association with the outcome variable for reasons beyond its effect on the endogenous regressor (exclusion restriction) and (3) does not share common causes with the outcome variable (independence). Finding instruments satisfying these conditions is typically difficult, however, thanks to our experiment, we can exploit the exogenous variation in the perceived trade-off induced by our treatments. More precisely, we take advantage of the fact that assignment to the treatments is random and can be used as an exogenous instrument predicting preferences for health-centered policies in the first-stage. In fact, given the random assignment of individuals to treatment conditions, exposure to our treatments is uncorrelated with the error term, then satisfying a crucial condition for a valid instrument. In addition, as shown in the previous section, the HP-EC and the HC-EP treatments produce a significant effect on the suspected endogenous variable *Health-Economy Trade-off*. As regards the exclusion restriction assumption, we rely on the idea that assignment to treatment does not produce any direct effect on compliance and all the impact on the outcome variable is mediated by the impact produced on individual preferences.

In Table 5 we report reduced form estimates. We estimate specifications (5) and (6) reported in Table 4, including the full set of controls and using alternatively the two outcome variables *Compliance PCA* and *Compliance*. In the first two columns, we add to the independent variables the HP-EC treatment, which produces a positive and statistically significant effect on both measures of compliance. In columns (3) and (4) we add also the HC-EP treatment. The effect of the HP-EC treatment is still positive and statistically significant while the HC-EP treatment does not produce any impact. Finally, in the last two columns we include all the treatments. We still find a positive impact of the HP-EC treatment on the outcome variables, but estimates are less precise.

**Table 5:** Reduced form estimates.

	Compliance PCA	Compliance	Compliance PCA	Compliance	Compliance PCA	Compliance
	(1)	(2)	(3)	(4)	(5)	(6)
HP-EC	0.1732**	7.7657***	0.1953**	9.2031***	0.1421	6.8263*
	(0.0751)	(3.2135)	(0.0799)	(3.4358)	(0.0920)	(3.9938)
HC-EP			0.0710	4.6167	0.0189	2.2884
			(0.1133)	(4.5784)	(0.1211)	(4.9660)
HP-EP					-0.1064	-4.7563
					(0.1052)	(4.4902)
CONTROLS	YES	YES	YES	YES	YES	YES
Observations	1836	1836	1836	1836	1836	1836

OLS Estimates.

Notes: Standard errors (corrected for heteroscedasticity) are reported in parentheses. The symbols ***, **, * indicate that the coefficients are statistically significant at the 1, 5 and 10 percent level, respectively.

Based also on the results shown in the previous table, in our preferred 2SLS specification we only use the HP-EC treatment as an instrument for the endogenous variable. However, we also experiment with alternative specifications adding as instruments the other treatments. Results of this analysis are reported in Table 6. We again estimate specifications (5) and (6) reported in Table 4, including the full set of controls and using alternatively the two outcome variables *Compliance* and *Compliance PCA*. In the first two columns, we use as an instrument the HP-EC treatment. First-stage regression results (Panel B) confirm a strong and significant effect of the treatment HP-EC on the perceived trade-off. First stage F-test statistics (32.17) is well above the common threshold of 10 used to detect weak instruments. Importantly, second stage regressions (Panel A) show a positive and statistically significant effect of the perceived trade-off on both measures of compliance.

**Table 6:** Intention to adhere to prescribed behaviors and health-economy preferences.

	Compliance PCA	Compliance	Compliance PCA	Compliance	Compliance PCA	Compliance
	(1)	(2)	(3)	(4)	(5)	(6)
	**Panel A**					
	**Second-stage regressions**					
$Tra\hat{de-}off$	0.8211**	36.8269**	0.6664*	28.1741*	0.6123*	25.7774*
	(0.3639)	(15.5436)	(0.3682)	(15.3565)	(0.3636)	(15.1808)
	**Panel B**					
	**First-stage regressions**					
HP-EC	0.2109***	0.2109***	0.1888***	0.1888***	0.2048***	0.2048***
	(0.0372)	(0.0372)	(0.0392)	(0.0392)	(0.0450)	(0.0450)
HC-EP			-0.0710*	-0.0710*	-0.0553	-0.0553
			(0.0376)	(0.0376)	(0.0437)	(0.0437)
HP-EP					0.0321	0.0321
					(0.04390)	(0.04390)
Kleibergen-Paap rk Wald F statistic	32.17	32.17	18.07	18.07	12.25	12.25
Hansen Test p_value			0.260	0.123	0.272	0.146
CONTROLS	YES	YES	YES	YES	YES	YES
Observations	1836	1836	1836	1836	1836	1836

IV Estimates.

Notes: Standard errors (corrected for heteroscedasticity) are reported in parentheses. The symbols ***, **, * indicate that the coefficients are statistically significant at the 1, 5 and 10 percent level, respectively. Controls included in both first-stage and second-stage regressions.

In columns (3) and (4) we use as instruments both HP-EC and HC-EP–the two treatments producing an effect on the endogenous variable–and find results in line with those discussed above. The Hansen test with a *p*-value of 0.260 and 0.123 in the two specifications indicates that the overidentifying restrictions are not rejected. F-statistics for the test that the coefficients of the instruments in the First Stage are jointly zero are again above the rule of thumb threshold suggested by [59] (18.07). Finally, in columns (5) and (6) in order to exploit all the variability in our data we use the all the treatments. The F-test is equal to 12.25 and the Hansen tests has a *p*-value of 0.272 and 0.146 in the two specifications. Again, results are qualitatively similar to those discussed above.

It worthwhile to notice that the magnitude of the impact produced by individual preferences (*Health-Economy Trade-off*) on the intention to adhere to prescribed behaviors changes in relation to the instrument used. This suggests that they are likely to generate different sets of compliers and, when we rely on all the sets of possible instruments, the estimated Local Average Treatment Effect (LATE) applies to a less specific group of individuals.

These results taken together further confirm that the type of communication we have analyzed in this paper affects intentions to adhere to prescribed behaviors through a switch in preferences over the health-economy trade-off.

## 6. Conclusions

The management of the health emergency by Covid-19 represents a great public challenge that requires a massive effort in terms of individual cooperation in order to limit the diffusion of the epidemic. The role of public communication, especially in the absence of financial incentives, has been recognized by several studies as decisive in order to ensure individual compliance with recommended behaviors. In particular, a key issue to be addressed concerns the management of the trade-off between public health and economic outcomes.

In this paper, we study how young individuals balance this trade-off during the pandemic and how the communication strategy over this trade-off affects their preferences for policies aimed at managing the restart of economic and social activities, and, ultimately their intention to adhere to prescribed behaviors. We investigate this issue in Italy—one of the country most affected by the outbreak—using a field experiment involving around 2000 students who took part in a survey administered during the period 20^th^ April - 25^th^ April, i.e. at the beginning of the Covid-19 pandemic and before the end of the first lockdown period. In our analysis we compare a positive framing which focuses on protective strategies (“protection”) with a negative framing focusing on potential losses (“costs”).

Our results show that a policy focusing on the protection of health and the costs for the worsening of the economic situation induces students to give more weight to health issues than when the trade-off is articulated in terms of costs for both health and economy. The effect is substantial, highly effective across different typologies of audiences, especially females, and associated with a higher intention to comply with precautionary behaviors. We find that 47.36% of students responded that they would consider ‘extremely’ or ‘very much’ health when framed as protection versus economic costs, while this share reduces to 34.15% in the group having both elements of the trade-off framed as costs.

These results pertaining to a specific group of individuals cannot be extended to the whole population. Nonetheless, as the behavior of young individuals can be crucial in order to control the spread of the infection, they have important policy implications. They suggest that the communication strategy during an emergency—such as that originating from the diffusion of the Covid-19 pandemic—plays a critical role and that a positive framing that focuses on the “protection” of the health conditions is likely to significantly affect individual preferences over the health dimension of the crisis. Under the assumption that self-reported preferences do not deviate significantly from real preferences, we may speculate that such a communication is likely to increase political consensus and may represent a costless strategy to ensure higher compliance with recommendations in the phases following the end of lockdown measures.

Being able to shape individual preferences over the health-economy trade-off, especially with cost-effective measures, is particularly important as such preferences affect individual decision to comply with behaviors that have been strongly recommended by doctors and specialists since the onset of the emergency in order to limit the spread of the virus. Exploiting the random assignment to the treatments in an instrumental variables framework, we provide causal evidence of a positive effect of health-oriented preferences on compliance. This suggests that an effective communication strategy may be a way to induce an otherwise non-incentivized active role in the defeat of the epidemic.

Moreover, our paper shows that characteristics such as personal attitudes, specific knowledge (i.e. the field of study) and state-dependent conditions affect preferences for the health-economy trade-off during the Covid-19 pandemic, regardless of the framing of the communication. Among these, the differences due to socio-economic background may pose important policy concerns. In many countries, current political debate is dominated by very polarized positions over the priorities to be given to the management of the reopening phase. Our paper suggests that the asymmetric economic consequences of the pandemic might explain these differences. One implication of this result is that the decision to financially help people who faced large economic shocks may also be supported as a way to strengthen social cohesion and preferences alignment over the management of the Covid-19 pandemic.

Finally, we find an interesting gender differential in the impact of the framing of the communication on preferences over the trade-off that might deserve further exploration. Despite the fact that when the survey was administered the health consequences of the Covid-19 virus seemed to be less pronounced among women, we find that they are significantly more affected by a positive framing focusing on the protection of the health conditions. Whether this depends on gender specific attitudes or on the role model of the male breadwinner might be a nice area of future research.

## Supporting information

S1 Appendix Survey proposed to students. (DOCX)

S2 Appendix Ordered logit and multinomial logit estimates. (DOCX)

S3 Appendix Heterogeneous impact of framing: Econometric analysis. (DOCX)

S1 Data (DTA)
