# Supplementary material for: The health-economy trade-off during the Covid-19 pandemic: Communication matters
Source: PLoS One. 2021 Sep 13;16(9):e0256103. doi: 10.1371/journal.pone.0256103 (PMC8437286; doi:10.1371/journal.pone.0256103)
Supplement: S1 Appendix — (DOCX) [file pone.0256103.s001.docx]

***Appendix A. Survey Proposed to Students***

The purpose of this research is to study individual behavior in response to the COVID-19 epidemic.

Completing the questionnaire takes less than 10 minutes. You will be asked to indicate your behavior in the coming months and your opinion on the policies that aim to decrease the spread of the virus. You will also be asked for personal or sensitive information that is important to the study results, some of which may make you feel anxious or embarrassed.

What do I need to know about a research project?

• The data is collected anonymously.

• Your participation is completely voluntary.

• You can decide not to be part of the project.

• This decision will not be used against you.

• You can ask any question you want before deciding.

Who can I talk to?

If you have any questions, concerns or complaints or if you think the research has damaged you, please contact Prof. Francesca Gioia at the e-mail address francesca.gioia@unical.it.

The information will be processed in compliance with privacy protection laws and used to publish summaries of the study results in academic journals, on the internet or at research conferences. You will not be directly identified in any publications or reports related to this study.

**Survey**

1. Department offering your degree program
2. Place of residence
3. Gender
4. Age
5. Is there anyone (including you) of your close acquaintance (relative, friend) who tested positive to COVID-19?
6. Some research shows that the closure of non-essential activities was accompanied in Italy by a reduction of Rt (an indicator of the spread of the epidemic) from 8.2 to 0.4. However, each week of non-essential business closures seems to reduce a country's income and profits by 0.75%. If you were the head of government and the following scenarios were proposed to you for the next two months, which one would you choose:

a) No closure, Rt = 8.2, Reduction of gross domestic product = 0%;

b) Closes ¼ of non-essential activities, Rt = 6.15, Gross domestic product reduction = 1.5%;

c) Half of non-essential activities closed, Rt = 4.1, Gross domestic product reduction = 3%;

d) All non-essential activities closed, Rt = 0.4, Gross domestic product reduction = 6%.

1. The government is planning the reopening after the temporary self-isolation measures introduced to deal with the coronavirus emergency. At this stage, it is necessary to consider the consequences that each decision has in terms of protection (costs for the worsening) of health - number of infections- and protection (costs for the worsening) of the economic situation. If you were the head of the government, which strategy would you choose?” The four combinations of A e B were also proposed in the question.
2. I would consider extremely the A of health and not much the B of the economic situation
3. I would consider very much the A of health and a little bit the B of the economic situation
4. I would consider enough the A of health and enough the B of the economic situation
5. I would consider a little bit the A of health and very much the B of the economic situation
6. I would consider not much the A of health and extremely the B of the economic situation
7. From 0 to 100, to what extent DO YOU INTEND TO FOLLOW the following behaviors after May 3rd? [0…100]
8. Stay at home as much as possible
9. Do not attend social events
10. Wear face mask when I have to go out
11. Stay at least 2 meters from other people
12. Wash your hands frequently
13. Staying home when sick
14. Avoid hugs and handshakes
15. To what extent do these statements correspond to how you are feeling now?
16. I am nervous when I think about the current situation
17. I am calm and relaxed
18. I am worried about my health
19. I am concerned about the health of my family
20. It stresses me out of the house
21. Over the past 2 weeks, how often have you bothered with each of the following issues?

[not at all, several days, more than half the days, and nearly every day]

1. Little interest or pleasure in doing things
2. Feeling down, sad or hopeless
3. Troubles falling or staying asleep or sleeping too much
4. Feeling tired or having little energy
5. Poor appetite or overeating
6. Feeling bad about yourself or that you are a failure or have let yourself or your family down
7. Trouble concentrating on things, such as reading the newspaper or watching television
8. Moving or speaking so slowly that other people could have noticed/ or the opposite being so fidgety or restless that you have been moving around a lot more than usual
9. To what extent do you think these characteristics describe you? I see myself as a person who is:
10. Extroverted, exuberant
11. Reliable, self-disciplined
12. Anxious, easily agitated
13. Selfless, who thinks a lot about others
14. Open to new experiences, with many interests
15. Disorganized, distracted
16. How many square meters is your home?
17. How many people are living in the house with you right now?
18. What is your father's educational qualification?
19. What is your mother's educational qualification?
20. From the beginning of the coronavirus emergency to today, is your father working?
21. No, he didn't even work before the emergency
22. No, due to the emergency but he receives income
23. No, due to the emergency and does not receive income
24. Yes, from home
25. Yes, keep going to work
26. From the beginning of the coronavirus emergency to today, is your mother working?
27. No, he didn't even work before the emergency
28. No, due to the emergency but he receives income
29. No, due to the emergency and does not receive income
30. Yes, from home
31. Yes, keep going to work
