# Supplementary material for: The health-economy trade-off during the Covid-19 pandemic: Communication matters
Source: PLoS One. 2021 Sep 13;16(9):e0256103. doi: 10.1371/journal.pone.0256103 (PMC8437286; doi:10.1371/journal.pone.0256103)
Supplement: S2 Appendix — (DOCX) [file pone.0256103.s002.docx]

***Appendix B: Ordered Logit and Multinomial Logit Estimates***

**Table B1. The impact of communication on preferences for policies aimed at managing the Covid-19 crisis. Ordered Logit Estimates**

|  | *Health-Economy Trade-off* | | | | | |
| --- | --- | --- | --- | --- | --- | --- |
|  | (1) | (2) | (3) | (4) | (5) | (6) |
| HP-EC | 0.5531*** | 0.5732*** | 0.5820*** | 0.6162*** | 0.6144*** | 0.6227*** |
|  | (0.1320) | (0.1320) | (0.1333) | (0.1366) | (0.1377) | (0.1381) |
| HC-EP | -0.1870 | -0.2246 | -0.2215 | -0.2220 | -0.2183 | -0.2092 |
|  | (0.1393) | (0.1433) | (0.1450) | (0.1469) | (0.1482) | (0.1483) |
| HP-EP | 0.0323 | 0.0504 | 0.0490 | 0.0651 | 0.0976 | 0.0926 |
|  | (0.1382) | (0.1380) | (0.1386) | (0.1402) | (0.1414) | (0.1421) |
| Female |  | -0.0456 | -0.0543 | -0.0295 | -0.1831 | -0.1880 |
|  |  | (0.1161) | (0.1164) | (0.1172) | (0.1268) | (0.1269) |
| Age |  | -0.0428* | -0.0445* | -0.0484** | -0.0517** | -0.0523** |
|  |  | (0.0240) | (0.0241) | (0.0241) | (0.0241) | (0.0241) |
| Sciences |  | 0.4318*** | 0.4313*** | 0.3644*** | 0.3871*** | 0.3897*** |
|  |  | (0.1219) | (0.1222) | (0.1251) | (0.1270) | (0.1273) |
| Humanities |  | 0.2104 | 0.2186 | 0.1641 | 0.1632 | 0.1514 |
|  |  | (0.1463) | (0.1473) | (0.1485) | (0.1501) | (0.1509) |
| Engineering |  | 0.1513 | 0.1209 | 0.0230 | 0.0539 | 0.0582 |
|  |  | (0.1522) | (0.1541) | (0.1569) | (0.1579) | (0.1585) |
| Parents’ Education |  |  | 0.0412*** | 0.0420*** | 0.0407*** | 0.0406*** |
|  |  |  | (0.0148) | (0.0150) | (0.0152) | (0.0152) |
| People/mq |  |  | 4.5677* | 4.5374* | 4.7326** | 4.5693* |
|  |  |  | (2.4003) | (2.3564) | (2.3911) | (2.4071) |
| Baseline Health-Economy Trade-off |  |  |  | 0.4493*** | 0.4365*** | 0.4371*** |
|  |  |  |  | (0.0671) | (0.0674) | (0.0675) |
| Parents Unemployed Covid-19 |  |  |  |  | -0.2107* | -0.2092* |
|  |  |  |  |  | (0.1127) | (0.1131) |
| Experienced Covid-19 |  |  |  |  | 0.0231 | 0.0332 |
|  |  |  |  |  | (0.1513) | (0.1524) |
| Anxiety severity scale |  |  |  |  | 0.0774*** | 0.0762*** |
|  |  |  |  |  | (0.0187) | (0.0191) |
| Depression severity scale |  |  |  |  | -0.0161 | -0.0193* |
|  |  |  |  |  | (0.0109) | (0.0110) |
| Altruist |  |  |  |  |  | 0.2618* |
|  |  |  |  |  |  | (0.1399) |
| Trustworthy |  |  |  |  |  | -0.1111 |
|  |  |  |  |  |  | (0.1198) |
| Extroverted |  |  |  |  |  | 0.0574 |
|  |  |  |  |  |  | (0.2263) |
| Open new experiences |  |  |  |  |  | -0.0009 |
|  |  |  |  |  |  | (0.1378) |
| Neurotic |  |  |  |  |  | 0.0198 |
|  |  |  |  |  |  | (0.1628) |
| Province of Residence FE | NO | NO | YES | YES | YES | YES |
| Observations | 1836 | 1836 | 1836 | 1836 | 1836 | 1836 |

Notes: Standard errors (corrected for heteroscedasticity) are reported in parentheses. The symbols ***, **, * indicate that the coefficients are statistically significant at the 1, 5 and 10 percent level, respectively.

**Table B2. The impact of communication on preferences for policies aimed at managing the Covid-19 crisis. Multinomial Logit Estimates**

|  | *Health-Economy Trade-off* | | | | | |
| --- | --- | --- | --- | --- | --- | --- |
|  | (1) | (2) | (3) | (4) | (5) | (6) |
|  |  |  |  |  |  |  |
| **I would consider not much the A of health and extremely the B of the economic situation** | | | | | | |
| HP-EC | -1.2287 | -1.0894 | -1.2698 | -0.7200 | -0.8932 | -1.8939 |
|  | (1.1217) | (1.0994) | (1.1506) | (1.3948) | (0.9152) | (1.8050) |
| HC-EP | -1.4727 | -1.5023 | -1.8191* | -1.1752 | -0.9294 | -2.8867* |
|  | (1.1213) | (1.1219) | (1.0392) | (1.2516) | (0.8634) | (1.5523) |
| HP-EP | -0.2946 | -0.2922 | -0.4076 | -0.0862 | 0.2984 | 0.4932 |
|  | (0.7685) | (0.8078) | (0.8588) | (0.9315) | (0.9816) | (1.5848) |
|  |  |  |  |  |  |  |
|  |  |  |  |  |  |  |
| **I would consider a little bit the A of health and very much the B of the economic situation** | | | | | | |
| HP-EC | -0.7587 | -0.8218 | -0.8025 | -0.8189 | -0.7999 | -0.7860 |
|  | (0.8414) | (0.8560) | (0.8535) | (0.8562) | (0.8414) | (0.8463) |
| HC-EP | 0.2500 | 0.5950 | 0.7284 | 0.7192 | 0.8447 | 0.9725 |
|  | (0.5914) | (0.6334) | (0.6719) | (0.6781) | (0.6539) | (0.6947) |
| HP-EP | -0.5177 | -0.6247 | -0.6350 | -0.6239 | -0.6807 | -0.6736 |
|  | (0.7352) | (0.7440) | (0.7433) | (0.7450) | (0.7872) | (0.7833) |
|  |  |  |  |  |  |  |
|  |  |  |  |  |  |  |
| **I would consider very much the A of health and a little bit the B of the economic situation** | | | | | | |
| HP-EC | 0.5143*** | 0.5445*** | 0.5456*** | 0.5758*** | 0.5784*** | 0.5833*** |
|  | (0.1478) | (0.1479) | (0.1497) | (0.1522) | (0.1532) | (0.1535) |
| HC-EP | -0.1471 | -0.1773 | -0.1876 | -0.1870 | -0.1822 | -0.1817 |
|  | (0.1549) | (0.1601) | (0.1621) | (0.1635) | (0.1645) | (0.1646) |
| HP-EP | 0.0097 | 0.0312 | 0.0251 | 0.0416 | 0.0730 | 0.0682 |
|  | (0.1536) | (0.1537) | (0.1546) | (0.1557) | (0.1567) | (0.1572) |
|  |  |  |  |  |  |  |
|  |  |  |  |  |  |  |
| **I would consider extremely the A of health and not much the B of the economic situation** | | | | | | |
| HP-EC | 0.5913** | 0.6030*** | 0.6399*** | 0.6995*** | 0.7394*** | 0.7641*** |
|  | (0.2338) | (0.2335) | (0.2358) | (0.2376) | (0.2375) | (0.2421) |
| HC-EP | -0.3837 | -0.4132 | -0.4001 | -0.4055 | -0.3924 | -0.3548 |
|  | (0.2715) | (0.2810) | (0.2845) | (0.2849) | (0.2864) | (0.2874) |
| HP-EP | -0.0069 | 0.0039 | 0.0097 | 0.0438 | 0.0887 | 0.0797 |
|  | (0.2532) | (0.2532) | (0.2573) | (0.2576) | (0.2580) | (0.2605) |
| Observations | 1836 | 1836 | 1836 | 1836 | 1836 | 1836 |

Notes: Standard errors (corrected for heteroscedasticity) are reported in parentheses. The symbols ***, **, * indicate that the coefficients are statistically significant at the 1, 5 and 10 percent level, respectively.
