# Supplementary material for: The health-economy trade-off during the Covid-19 pandemic: Communication matters
Source: PLoS One. 2021 Sep 13;16(9):e0256103. doi: 10.1371/journal.pone.0256103 (PMC8437286; doi:10.1371/journal.pone.0256103)

***Appendix C: Heterogeneous impact of framing: econometric analysis***

**Table C1: Predetermined characteristics and background**

|  | Males | Females | Below Avg Age | Above Avg Age | Low P Edu | High P Edu |
| --- | --- | --- | --- | --- | --- | --- |
|  | (1) | (2) | (3) | (4) | (5) | (6) |
| HP-EC | 0.0562 | 0.2791*** | 0.2785*** | 0.1583*** | 0.1327** | 0.2529*** |
|  | (0.0826) | (0.0507) | (0.0653) | (0.0585) | (0.0647) | (0.0590) |
| HC-EP | -0.1056 | -0.0360 | -0.0252 | -0.0735 | -0.1145* | -0.0303 |
|  | (0.0867) | (0.0530) | (0.0655) | (0.0613) | (0.0658) | (0.0627) |
| HP-EP | 0.0499 | 0.0507 | 0.0572 | 0.0213 | -0.0734 | 0.1220** |
|  | (0.0861) | (0.0510) | (0.0661) | (0.0601) | (0.0643) | (0.0604) |
| CONTROLS | YES | YES | YES | YES | YES | YES |
| Observations | 535 | 1301 | 824 | 1012 | 886 | 950 |
|  |  |  |  |  |  |  |
| Test HP-EC | 0.023 | | 0.180 | | 0.177 | |
| Test HC-EP | 0.462 | | 0.576 | | 0.334 | |
| Test HP-EP | 0.994 | | 0.682 | | 0.024 | |

Notes: Standard errors (corrected for heteroscedasticity) are reported in parentheses. The symbols ***, **, * indicate that the coefficients are statistically significant at the 1, 5 and 10 percent level, respectively.

**Table C2: Personality traits**

|  | Altruist | | Trustworthy | | Extroverted | |
| --- | --- | --- | --- | --- | --- | --- |
|  | No | Yes | No | Yes | No | Yes |
|  | (1) | (2) | (3) | (4) | (5) | (6) |
| HP-EC | 0.1615*** | 0.3931*** | 0.1477*** | 0.3738*** | 0.2061*** | 0.3802 |
|  | (0.0476) | (0.1013) | (0.0512) | (0.0826) | (0.0440) | (0.2631) |
| HC-EP | -0.0664 | 0.0264 | -0.1159** | 0.0848 | -0.0640 | 0.1146 |
|  | (0.0487) | (0.1085) | (0.0526) | (0.0847) | (0.0454) | (0.2436) |
| HP-EP | 0.0254 | 0.0684 | -0.0115 | 0.1650* | 0.0425 | 0.0232 |
|  | (0.0486) | (0.0993) | (0.0510) | (0.0868) | (0.0446) | (0.2567) |
| CONTROLS | YES | YES | YES | YES | YES | YES |
| Observations | 1442 | 394 | 1308 | 528 | 1733 | 103 |
|  |  |  |  |  |  |  |
| Test HP-EC | 0.034 | | 0.023 | | 0.501 | |
| Test HC-EP | 0.387 | | 0.033 | | 0.458 | |
| Test HP-EP | 0.682 | | 0.059 | | 0.938 | |

Notes: Standard errors (corrected for heteroscedasticity) are reported in parentheses. The symbols ***, **, * indicate that the coefficients are statistically significant at the 1, 5 and 10 percent level, respectively.

**Table C3: Covid-19 health and economic implications**

|  | Experienced Covid-19 | | Parents Unemployed | | Depression Severity Scale | | Anxiety Severity Scale | |
| --- | --- | --- | --- | --- | --- | --- | --- | --- |
|  | No | Yes | No | Yes | Low | High | Low | High |
|  | (1) | (2) | (3) | (4) | (5) | (6) | (7) | (8) |
| HP-EC | 0.2098*** | 0.2444* | 0.1721*** | 0.2623*** | 0.1686*** | 0.2611*** | 0.1808*** | 0.2224*** |
|  | (0.0460) | (0.1303) | (0.0512) | (0.0867) | (0.0577) | (0.0663) | (0.0611) | (0.0620) |
| HC-EP | -0.0765 | 0.0977 | -0.0573 | -0.0524 | -0.0445 | -0.0765 | -0.0441 | -0.0699 |
|  | (0.0470) | (0.1376) | (0.0532) | (0.0870) | (0.0601) | (0.0668) | (0.0623) | (0.0646) |
| HP-EP | 0.0299 | 0.0918 | 0.0207 | 0.0350 | -0.0116 | 0.0859 | 0.0274 | 0.0415 |
|  | (0.0466) | (0.1300) | (0.0527) | (0.0845) | (0.0612) | (0.0640) | (0.0617) | (0.0627) |
| CONTROLS | YES | YES | YES | YES | YES | YES | YES | YES |
| Observations | 1598 | 238 | 1329 | 507 | 919 | 917 | 925 | 911 |
|  |  |  |  |  |  |  |  |  |
| Test HP-EC | 0.794 | | 0.411 | | 0.298 | | 0.642 | |
| Test HC-EP | 0.234 | | 0.803 | | 0.710 | | 0.767 | |
| Test HP-EP | 0.636 | | 0.988 | | 0.260 | | 0.873 | |

Notes: Standard errors (corrected for heteroscedasticity) are reported in parentheses. The symbols ***, **, * indicate that the coefficients are statistically significant at the 1, 5 and 10 percent level, respectively.

**Table C4: Baseline preferences**

|  | Economic Oriented | Middle | Health Oriented |
| --- | --- | --- | --- |
|  | (1) | (2) | (3) |
| HP-EC | 0.2697*** | 0.1955*** | 0.1519* |
|  | (0.0946) | (0.0630) | (0.0816) |
| HC-EP | -0.1170 | -0.0577 | -0.0611 |
|  | (0.0926) | (0.0637) | (0.0798) |
| HP-EP | 0.0002 | -0.0163 | 0.1017 |
|  | (0.0998) | (0.0586) | (0.0828) |
| CONTROLS | YES | YES | YES |
| Observations | 397 | 810 | 629 |
|  |  |  |  |
| Test HP-EC (1)=(2) | 0.502 |  |  |
| Test HC-EP (1)=(2) | 0.588 |  |  |
| Test HP-EP (1)=(2) | 0.883 |  |  |
|  |  |  |  |
| Test HP-EC (1)=(3) | 0.333 |  |  |
| Test HC-EP (1)=(3) | 0.639 |  |  |
| Test HP-EP (1)=(3) | 0.421 |  |  |
|  |  |  |  |
| Test HP-EC (2)=(3) | 0.666 |  |  |
| Test HC-EP (2)=(3) | 0.973 |  |  |
| Test HP-EP (2)=(3) | 0.236 |  |  |

Notes: Standard errors (corrected for heteroscedasticity) are reported in parentheses. The symbols ***, **, * indicate that the coefficients are statistically significant at the 1, 5 and 10 percent level, respectively.

**Table C5: Predetermined characteristics and background with interaction**

|  | Gender | Age | Parents’ Education |
| --- | --- | --- | --- |
| HP-EC | 0.0449 | ^+++^0.4544*** | 0.2557** |
|  | (0.1442) | (0.1152) | (0.1176) |
| HC-EP | -0.2829* | -0.0374 | -0.2453** |
|  | (0.1500) | (0.1153) | (0.1194) |
| HP-EP | -0.0365 | 0.0779 | -0.1174 |
|  | (0.1553) | (0.1132) | (0.1190) |
| HP-EC * Female | ^++^0.4608*** |  |  |
|  | (0.1717) |  |  |
| HC-EP * Female | 0.2428 |  |  |
|  | (0.1774) |  |  |
| HP-EP * Female | 0.1398 |  |  |
|  | (0.1819) |  |  |
| HP-EC * Above Avg Age |  | -0.1572 |  |
|  |  | (0.1561) |  |
| HC-EP * Above Avg Age |  | -0.1357 |  |
|  |  | (0.1602) |  |
| HP-EP * Above Avg Age |  | -0.0350 |  |
|  |  | (0.1609) |  |
| HP-EC * High Parents’ Education |  |  | 0.1920 |
|  |  |  | (0.1570) |
| HC-EP * High Parents’ Education |  |  | 0.2350 |
|  |  |  | (0.1594) |
| HP-EP * High P Parents’ Education |  |  | 0.3217** |
|  |  |  | (0.1607) |
| Female | ^+^-0.3089** | -0.0888 | -0.1018 |
|  | (0.1295) | (0.0729) | (0.0717) |
| Above Avg Age |  | -0.0729 |  |
|  |  | (0.1143) |  |
| High Parents’ Education |  |  | -0.0994 |
|  |  |  | (0.1154) |
| CONTROLS | YES | YES | YES |
| Observations | 1836 | 1836 | 1836 |

Notes: Standard errors (corrected for heteroscedasticity) are reported in parentheses. The symbols ***, **, * indicate that the coefficients are statistically significant at the 1, 5 and 10 percent level, respectively. The symbols ^+++^, ^++^, ^+^ indicate that the coefficients are statistically significant at the 1, 5 and 10 percent level, respectively, when applying the Sidak’s and the Holm’s method for adjusting p-values for multiple testing.

**Table C6: Personality traits with interaction**

|  | Altruist | Trustworthy | Extroverted |
| --- | --- | --- | --- |
| HP-EC | ^+++^0.2900*** | ^++^0.2537*** | ^+++^0.3645*** |
|  | (0.0882) | (0.0916) | (0.0802) |
| HC-EP | -0.1350 | ^++^-0.2415** | -0.1339 |
|  | (0.0904) | (0.0958) | (0.0838) |
| HP-EP | 0.0337 | -0.0358 | 0.0736 |
|  | (0.0907) | (0.0936) | (0.0826) |
| HP-EC * Altruist | 0.3526* |  |  |
|  | (0.1931) |  |  |
| HC-EP * Altruist | 0.0841 |  |  |
|  | (0.2050) |  |  |
| HP-EP * Altruist | 0.1081 |  |  |
|  | (0.1965) |  |  |
| HP-EC * Trustworthy |  | ^+^0.3988** |  |
|  |  | (0.1764) |  |
| HC-EP * Trustworthy |  | ^+^0.4417** |  |
|  |  | (0.1815) |  |
| HP-EP * Trustworthy |  | 0.3426* |  |
|  |  | (0.1821) |  |
| HP-EC * Extroverted |  |  | 0.0415 |
|  |  |  | (0.4063) |
| HC-EP * Extroverted |  |  | 0.2962 |
|  |  |  | (0.4175) |
| HP-EP * Extroverted |  |  | -0.2924 |
|  |  |  | (0.3995) |
| Altruist | 0.0081 | 0.1514* | 0.1445* |
|  | (0.1455) | (0.0796) | (0.0795) |
| Trustworthy | -0.0581 | ^++^-0.3590*** | -0.0638 |
|  | (0.0672) | (0.1342) | (0.0672) |
| Extroverted | 0.0539 | 0.0514 | 0.0187 |
|  | 0.0081 | 0.1514* | 0.1445* |
| CONTROLS | YES | YES | YES |
| Observations | 1836 | 1836 | 1836 |

Notes: Standard errors (corrected for heteroscedasticity) are reported in parentheses. The symbols ***, **, * indicate that the coefficients are statistically significant at the 1, 5 and 10 percent level, respectively. The symbols ^+++^, ^++^, ^+^ indicate that the coefficients are statistically significant at the 1, 5 and 10 percent level, respectively, when applying the Sidak’s and the Holm’s method for adjusting p-values for multiple testing.

**Table C7: Covid-19 health and economic implications with interaction**

|  | Experienced  Covid-19 | Parents Unemployed | Depression Severity Scale | Anxiety Severity Scale |
| --- | --- | --- | --- | --- |
| HP-EC | ^+++^0.3640*** | ^+++^0.3246*** | ^++^0.2934*** | ^++^0.3251*** |
|  | (0.0848) | (0.0909) | (0.1022) | (0.1141) |
| HC-EP | -0.1605* | -0.0824 | -0.1110 | -0.1169 |
|  | (0.0862) | (0.0932) | (0.1096) | (0.1154) |
| HP-EP | 0.0415 | 0.0541 | -0.0423 | 0.0341 |
|  | (0.0868) | (0.0961) | (0.1101) | (0.1152) |
| HP-EC * Exp Covid-19 | 0.0051 |  |  |  |
|  | (0.2298) |  |  |  |
| HC-EP * Exp Covid-19 | 0.3543 |  |  |  |
|  | (0.2572) |  |  |  |
| HP-EP * Exp Covid-19 | 0.1102 |  |  |  |
|  | (0.2437) |  |  |  |
| HP-EC * Parents Unempl |  | 0.1661 |  |  |
|  |  | (0.1763) |  |  |
| HC-EP * Parents Unempl |  | -0.1089 |  |  |
|  |  | (0.1829) |  |  |
| HP-EP * Parents Unempl |  | 0.0145 |  |  |
|  |  | (0.1758) |  |  |
| HP-EC * Depression Sev Sc |  |  | 0.1538 |  |
|  |  |  | (0.1556) |  |
| HC-EP * Depression Sev Sc |  |  | -0.0083 |  |
|  |  |  | (0.1582) |  |
| HP-EP * Depression Sev Sc |  |  | 0.1887 |  |
|  |  |  | (0.1607) |  |
| HP-EC * Anxiety Sev Sc |  |  |  | 0.0736 |
|  |  |  |  | (0.1569) |
| HC-EP * Anxiety Sev Sc |  |  |  | 0.0063 |
|  |  |  |  | (0.1597) |
| HP-EP * Anxiety Sev Sc |  |  |  | 0.0277 |
|  |  |  |  | (0.1617) |
| Experienced Covid-19 | -0.0616 | 0.0479 | 0.0589 | 0.0500 |
|  | (0.1734) | (0.0850) | (0.0853) | (0.0855) |
| Parents Unemployed | -0.1263** | -0.1468 | -0.1328** | -0.1289** |
|  | (0.0635) | (0.1298) | (0.0633) | (0.0634) |
| Depression Sev Scale |  |  | -0.1432 |  |
|  |  |  | (0.1164) |  |
| Anxiety Sev Scale |  |  |  | 0.1564 |
|  |  |  |  | (0.1184) |
| CONTROLS | YES | YES | YES | YES |
| Observations | 1836 | 1836 | 1836 | 1836 |

Notes: Standard errors (corrected for heteroscedasticity) are reported in parentheses. The symbols ***, **, * indicate that the coefficients are statistically significant at the 1, 5 and 10 percent level, respectively. The symbols ^+++^, ^++^, ^+^ indicate that the coefficients are statistically significant at the 1, 5 and 10 percent level, respectively, when applying the Sidak’s and the Holm’s method for adjusting p-values for multiple testing.

**Table C8: Baseline preferences with interaction**

|  | Anxiety Severity Scale |
| --- | --- |
| HP-EC | ^++^0.5157*** |
|  | (0.1787) |
| HC-EP | -0.1576 |
|  | (0.1833) |
| HP-EP | 0.0110 |
|  | (0.1978) |
| HP-EC * Middle | -0.1363 |
|  | (0.2108) |
| HC-EP * Middle | 0.0744 |
|  | (0.2175) |
| HP-EP * Middle | -0.0218 |
|  | (0.2282) |
| HP-EC * Health Oriented | -0.2559 |
|  | (0.2222) |
| HC-EP * Health Oriented | 0.0287 |
|  | (0.2254) |
| HP-EP * Health Oriented | 0.1676 |
|  | (0.2400) |
| Middle | 0.1528 |
|  | (0.1579) |
| Health Oriented | 0.5023*** |
|  | (0.1645) |
| CONTROLS | YES |
| Observations | 1836 |

Notes: Standard errors (corrected for heteroscedasticity) are reported in parentheses. The symbols ***, **, * indicate that the coefficients are statistically significant at the 1, 5 and 10 percent level, respectively. The symbols ^+++^, ^++^, ^+^ indicate that the coefficients are statistically significant at the 1, 5 and 10 percent level, respectively, when applying the Sidak’s and the Holm’s method for adjusting p-values for multiple testing.

**Figure C1: Predetermined characteristics and background, distributions**


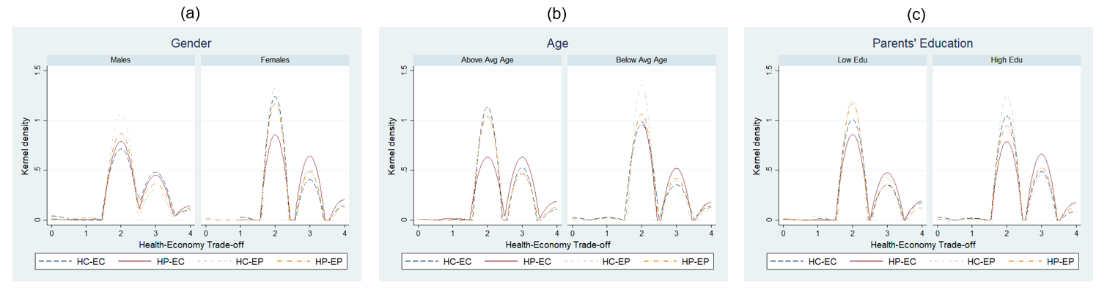


**Figure C2: Personality traits, distributions**


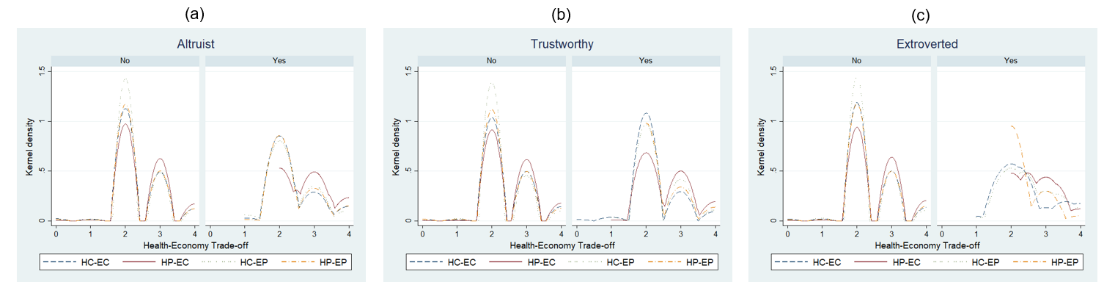


**Figure C3: Covid-19 health and economic implications, distributions**


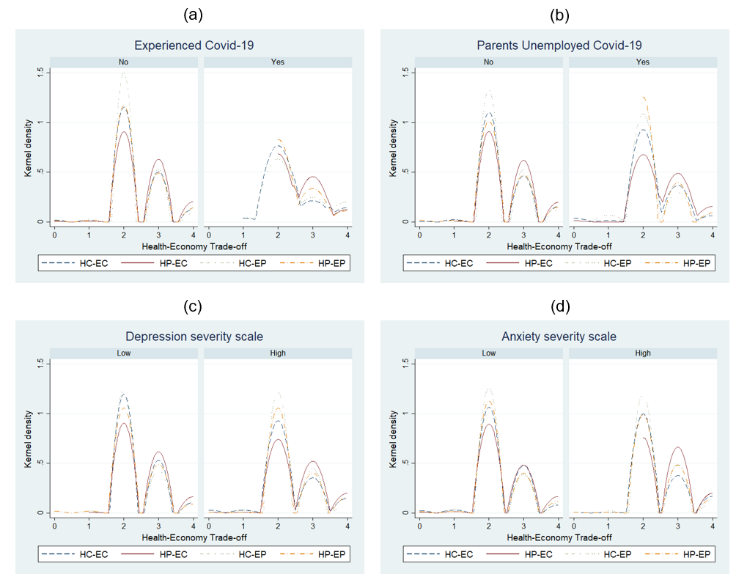


**Figure C4: Baseline preferences, distributions**


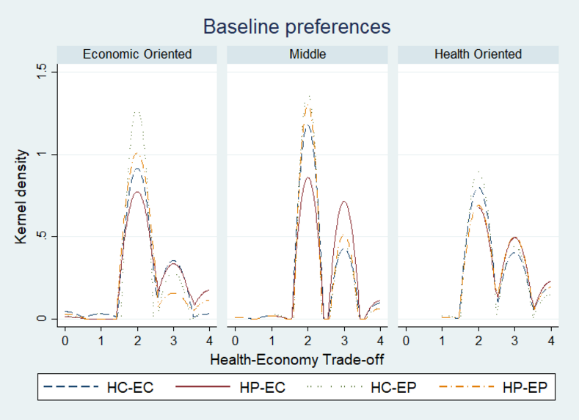

Supplement: S3 Appendix — (DOCX) [file pone.0256103.s003.docx]
